# Identification of 1H-purine-2,6-dione derivative as a potential SARS-CoV-2 main protease inhibitor: molecular docking, dynamic simulations, and energy calculations

**DOI:** 10.7717/peerj.14120

**Published:** 2022-10-07

**Authors:** Hossam Nada, Ahmed Elkamhawy, Kyeong Lee

**Affiliations:** 1 BK21 FOUR Team and Integrated Research Institute for Drug Development, College of Pharmacy, Dongguk University-Seoul, Goyang, South Korea; 2 Department of Pharmaceutical Chemistry, Faculty of Pharmacy, Badr University in Cairo, Cairo, Egypt; 3 Department of Pharmaceutical Organic Chemistry, Faculty of Pharmacy, Mansoura University, Mansoura, Egypt

**Keywords:** E-pharmacophore modeling, Structure-based virtual screening (SBVS), Molecular dynamics (MD) simulation, SARS-Cov-2 M*pro*, Free energy calculations

## Abstract

The rapid spread of the coronavirus since its first appearance in 2019 has taken the world by surprise, challenging the global economy, and putting pressure on healthcare systems across the world. The introduction of preventive vaccines only managed to slow the rising death rates worldwide, illuminating the pressing need for developing effective antiviral therapeutics. The traditional route of drug discovery has been known to require years which the world does not currently have. *In silico* approaches in drug design have shown promising results over the last decade, helping to decrease the required time for drug development. One of the vital non-structural proteins that are essential to viral replication and transcription is the SARS-CoV-2 main protease (Mpro). Herein, using a test set of recently identified COVID-19 inhibitors, a pharmacophore was developed to screen 20 million drug-like compounds obtained from a freely accessible Zinc database. The generated hits were ranked using a structure based virtual screening technique (SBVS), and the top hits were subjected to in-depth molecular docking studies and MM-GBSA calculations over SARS-COV-2 Mpro. Finally, the most promising hit, compound (**1**), and the potent standard (**III**) were subjected to 100 ns molecular dynamics (MD) simulations and *in silico* ADME study. The result of the MD analysis as well as the *in silico* pharmacokinetic study reveal compound **1** to be a promising SARS-Cov-2 MPro inhibitor suitable for further development.

## Introduction

Since the appearance of the coronavirus (SARS-CoV-2) in Wuhan City of China and its subsequent rapid spread to the rest of the world, it has caused a global health emergency due to its highly contagious nature with millions of people dying around the globe (Su et al., 2020). Even though effective vaccines have emerged, the increasing daily death toll shows the urgent need in developing new effective therapeutic drugs capable of treating the SARS-CoV-2 infection (Rabaan et al., 2020). Coronaviruses (CoVs) are a member of the Coronaviridae family which is the largest family of viruses triggering respiratory and gastrointestinal infections (Payne, 2017). The SARS-CoV-2 is largely airborne transmitted through respiratory droplets with an infection range of less than 2 m (Klompas, Baker & Rhee, 2020). It is a single-stranded RNA virus with two major groups of proteins: structural and non-structural proteins (Pal et al., 2020). Since there is no cure to date, both groups of proteins are being extensively investigated as prospective targets for developing novel drugs able to safely and potently inhibit SARS-CoV-2 (Wu et al., 2020).

**Figure 1:**
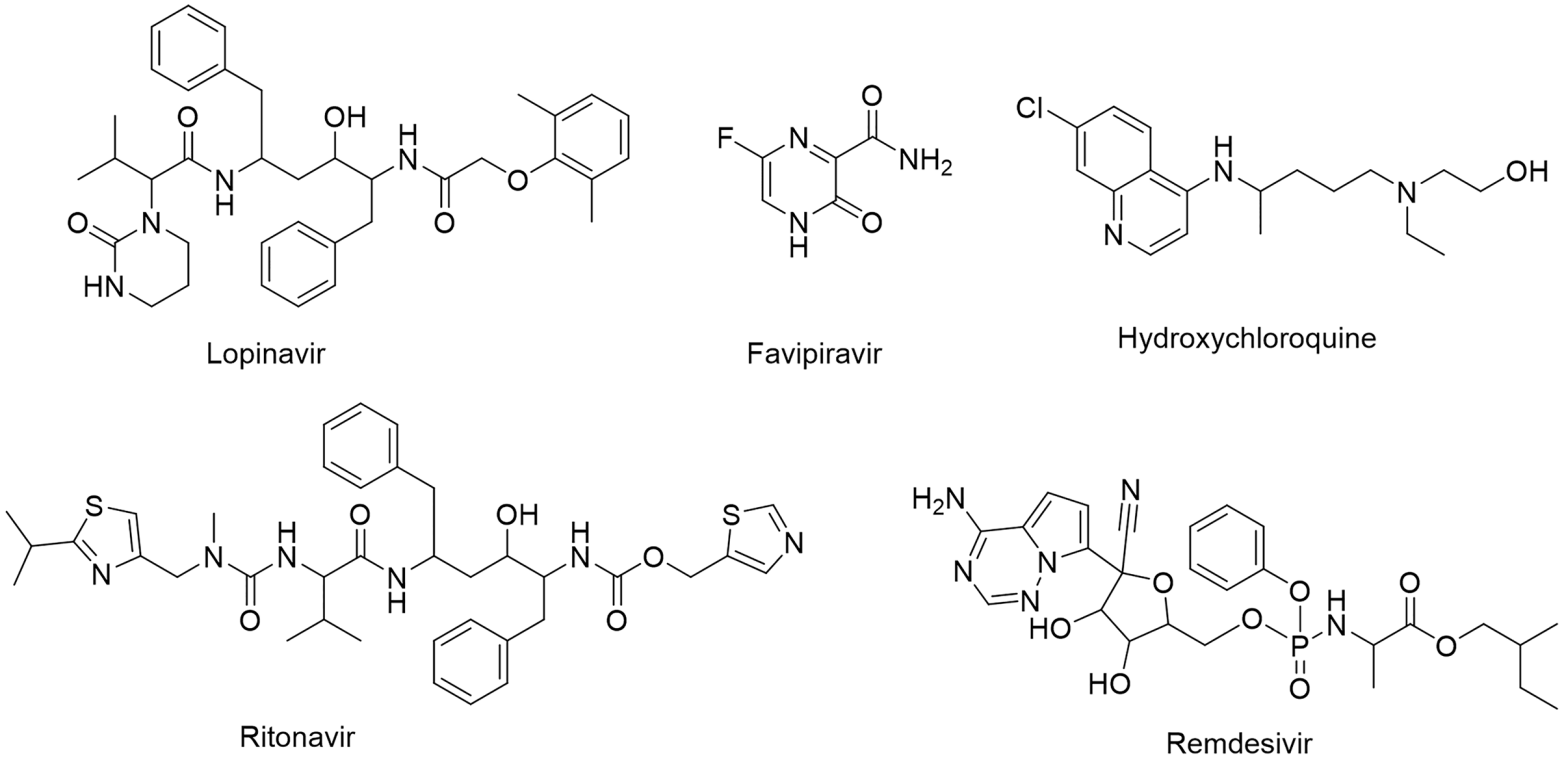
Chemical structures of FDA-approved drugs repurposed as potential therapeutic drugs for COVID-19.

One of the vital non-structural proteins to the viral replication and transcription is the SARS-CoV-2 M^pro^ which acts by proteolytically cutting the overlapping pp1a and pp1ab polyproteins (Cavasotto, Lamas & Maggini, 2021; Mohammad et al., 2020). SARS-CoV-2 M^pro^ is a promising target for the development of lead compounds to combat the COVID-19 pandemic (Naidoo et al., 2021). Various attempts have been employed to repurpose some FDA-approved drugs for COVID-19 treatment including lopinavir, favipiravir, hydroxychloroquine, ritonavir, and remdesivir (Fig. 1) (Drożdżal et al., 2020).

However, the clinical outcomes of these attempts have been inconsistent, showing the need for developing new candidate(s) with reliable potency and specificity (Miyamoto et al., 2012). Employing the traditional route of rationally synthesizing a large number of molecules and testing them is a lengthy and costly process (Choudhury, Arul Murugan & Priyakumar, 2022). On the other hand, *in silico* analysis is regarded as a solution for the lengthy drug development process through its ability to screen a large number of small molecules to identify new potential candidates for treatment of emerging diseases (Bharti & Shukla, 2021).

In this study, based on a validated pharmacophore developed from one of the potent SARS-COV2 inhibitors (compound **III** (Table S1) chosen for its reported potent inhibitory (inhibitory constant (K_i_) of 4.1 nM and an inhibitory concentration (IC_50_) of 1.7 μM (Gao et al., 2021; Tian et al., 2021)), we report a hybrid application of e-pharmacophore-based virtual screening and structure-based virtual screening (SBVS) strategies with the prime goal of identifying promising novel potential inhibitor(s) for SARS-CoV-2, utilizing integrated SBVS, molecular docking, MD simulations, MM-GBSA calculations, and *in silico* ADME approaches.

## Materials and Methods

The Schrodinger suite 2021 was used to prepare the protein, grid generation, pharmacophore Phase screening virtual screening, MM-GBSA scoring, and molecular docking against the SARS-CoV-2 M^pro^ (Schrodinger, Inc, New York, USA). For MD simulation studies, the GROMACS 2022.2 program was employed. Discovery studio visualizer, XMGRACE, and Visual Molecular Dynamics (VMD) tools were used to visualize the findings of the docking and MD simulations (Daina, Michielin & Zoete, 2017; Mysinger et al., 2012; Studio, 2008; Turner & Land-Margin Research OGIoS, and Technology B, OR, 2005).

### Protein preparation

The protein data bank was used to retrieve the crystal structure of SARS-COV-2 M^pro^ in complex with a novel inhibitor at a resolution of 2.2 Å (PDB: 6M2N) (Su et al., 2020). To check the validity of the downloaded crystal structures, the PROCHECK server was utilized to construct a Ramachandran plot (Laskowski et al., 1996). The validated PDB structure was prepared using the Protein Preparation Wizard module of Maestro, which included the removal of water molecules as well as the addition of any missing residues and hydrogen atoms. The prepared protein structure was protonated at physiological pH. Energy minimization was accomplished *via* a conjugate gradient strategy based on the OPLS-2005 force field, which yielded a unique low minimum of the structure (Stortz et al., 2009).

### Ligand preparation

The chemical library, comprising 20 million compounds (the “now” chemical library), was acquired from the freely accessible Zinc server (Sterling & Irwin, 2015). The following steps were taken to prepare the ligands: (i) The downloaded 3D structures of the ligands were imported into the Maestro program and optimized using the molecular mechanical (MM) geometry optimization approach. (ii) The ligands were adjusted to physiological pH (7.4) and geometry optimization was performed. (iii) The ligands were then subjected to energy minimization utilizing Maestro’s Ligprep module and the OPLS3 force field (Madhavi Sastry et al., 2013).

### Generation of pharmacophore hypothesis

A number of small molecules that demonstrated significant activity against SARS-CoV-2 were identified in a recent review which assessed the recent emerging small-molecule therapeutic options for COVID-19 and their different modes of action (Tian et al., 2021). These molecules were used to create a test set composed of 30 compounds (Table S1). A structure-based hypothesis and ligand-protein complex-based hypotheses were developed and validated following the same protocol as described in our previous investigation in identifying novel DDR1inhibitors (Nada et al., 2022b).

### E-pharmacophore-based virtual screening

Employing the Zinc database, a chemical library “now” was downloaded and prepared, then screened using the Maestro pharmacophore phase module (Sterling & Irwin, 2015). To be recognized as potential SARS-CoV-2 inhibitors, the screened compounds had to match five of the seven pharmacophoric features in the pharmacophore hypothesis. The database hits were ranked using a fitness score based on their RMSD, with a distance matching tolerance of 2.0 Å. SBVS was used to rank the hits that were recognized by the pharmacophore phase screening.

### Structure based virtual screening (SBVS)

SBVS was applied to rank the generated hits from the pharmacophore phase screening according to their binding affinity and GlideScores (Grid-based Ligand Docking with Energetics). SBVS uses a hierarchical series of filters to find feasible ligand sites in the active-site area of the receptor. The shape and features of the receptor are represented on a grid by multiple separate sets of fields to give progressively increasingly accurate scoring of the ligand poses (Jain & Ghate, 2014). GLIDE manages conformational flexibility in SBVS mode *via* conducting a thorough conformational search followed by a rapid exclusion of the undesirable conformations (Nada et al., 2022b).

### Molecular docking study

A molecular docking study was carried out to identify the compounds with the highest potential from the pharmacophore Phase screening *via* predicting their potential binding modes to SARS-CoV-2 M^pro^. To rule out any false positives, the top 30 SBVS candidates were docked into their own binding sites using the extra precision module of Glide, yielding 32 poses for each ligand. Compounds with a GlideScore higher than the standard, compound **III** (GlideScore = −8.12 Kcal/mol), were subjected to an in-depth molecular docking analysis. The docking figures were created with the BIOVIA Discovery Studio Visualizer 2020 package, and the docking scores were ranked, choosing the most negative scores for each complex.

### Pre-dynamic molecular mechanics-generalized born surface area (MM-GBSA) calculations

The binding free energy of the observed protein-ligand complexes was measured using the MM-GBSA approach, which integrates molecular mechanics (MM) force fields with a generalized Born and surface area continuum (implicit) solvation solvent model. The MM-GBSA calculation incorporates the OPLS-2005 force field, the VSGB solvent model, and the rotamer search algorithms (Greenidge et al., 2013; Li et al., 2011). The MM-GBSA was determined in this study using the Prime module of the Schrodinger suite (Prime, Schrödinger, LLC, New York, NY, USA) according to the equation below:

ΔG = E_complex (minimized) – [E_ligand (minimized) + E_receptor (minimized)]

The default setting employed to compute MM-GBSA involved rendering all protein atoms rigid while the ligand atoms are relaxed.

### Molecular dynamic (MD) simulations

The standard compound (**III**) and the top hit compound **1** bound to the SARS-CoV-2 M^pro^ were subjected to 100 ns molecular dynamics (MD) study to validate and support the ability of the designed system to identify potential SARS-CoV-2 inhibitors. The molecular dynamic simulations for compound **1** and the standard (**III**) were repeated once to ensure the reproducibility of the results. The MD simulations were performed using the GROningen MAchine for Chemical Simulations (GROMACS 2022.2) (Abraham et al., 2015). In the MD simulation, a monomeric chain (chain D) of the SARS-COV-2 M^pro^ (PDB: 6M2N) was used (Lokhande et al., 2021). The CHARMM22 forcefield was used to construct the MD systems for each simulation. Ligand topology was generated employing the freely accessible webservice, SwissParam (Zoete et al., 2011). The system consisted of a cubic box solvated with TIP3P water molecules and automatically neutralized with four Na^+^ ions generated using the “gmx genion -s instep -o solv_ions.gro -p tocolytic -pname NA -nname CL -neutral” script of GROMACS (Nada et al., 2022b). The steepest descent minimization approach was used to minimize the energy in two steps: thermalization at 300 K (in the NVT ensemble) and pressurization at 1 bar for 100 ps (in the NPT ensemble) using Berendsen thermostat and barostat (Leonis et al., 2012; Nada et al., 2022a). Only the solvent molecules were allowed to move unrestrictedly during both equilibrations to ensure proper equilibration of the system. The long-range electrostatics were determined utilizing particle mesh “Eshwald method” with a 1.2 nm (12 Å) cut-off and a 0.16 nm (1.6 Å) Fourier spacing approach (Dhankhar et al., 2020). A total of 100 ns of unrestricted production runs were performed on each of the four systems that had been re-calibrated.

VMD software was used to analyze the percentage of occupancy of each residue contributing to a hydrogen bond with the investigated inhibitors throughout the MD simulation (Humphrey, Dalke & Schulten, 1996; Salmas, Yurtsever & Durdagi, 2015). Only residues contributing to more than 10% of hydrogen bond occupancy were considered. The changes in distance of the compounds relative to the hydrogen bond contributing residues was calculated using the “gmx mindist” command of GROMACS (Jiang et al., 2021). The mass-weighted covariance matrix (C) of the atomic positional fluctuations is employed in highlighting the high amplitude, concerted motions of MD trajectories of the eigen vectors, in a calculation known as the principal component analysis (PCA) (Gurung & Bhattacharjee, 2018).

Solvent accessible surface area (SASA) analysis of the two complexes was carried out using the “gmx sasa” command of GROMCAS. PCA was calculated employing the “gmx covar” and “gmx anaeig” functions of GROMACS and the XMGRACE software was used to visualize the results. Gibbs free energy landscapes (FELs) calculations were calculated by utilizing the “g_sham” function of GROMACS using the predicted distribution probability of the top two principal components (PCs) (Mittal et al., 2021).

### Molecular mechanics Poisson–Boltzmann surface area (MM-PBSA) calculations

MM-PBSA is technique for calculating the interaction free energies of protein-ligand complexes during the molecular dynamic simulation (Anbarasu & Jayanthi, 2018). The trajectories of the MD simulations was retrieved and processed utilizing the open source g_mmpbsa package executed through GROMACS (Kumari, Kumar & Lynn, 2014). “g_mmpbsa” package implements the MM-PBSA methodology utilizing Gromacs and Adaptive Poisson-Boltzmann Solver package subroutines. “g_mmpbsa” only determines the electrostatic and Van der Waals components of gas-phase energy because the bonded contribution in the single-trajectory approach is zero. For the final 1 ns of each Md simulation, the energy components of each complex were computed (*i.e*. from 99 to 100 ns) (Jusoh et al., 2018).

### *In silico* pharmacokinetic study

The *in silico* pharmacokinetic study was conducted using the freely accessible Swiss ADME server (Daina, Michielin & Zoete, 2017) following the same methodology as reported in our previous investigation of multikinase inhibitors (Elsherbeny et al., 2021).

## Results

### Generation of the e-pharmacophore hypotheses

**Figure 2:**
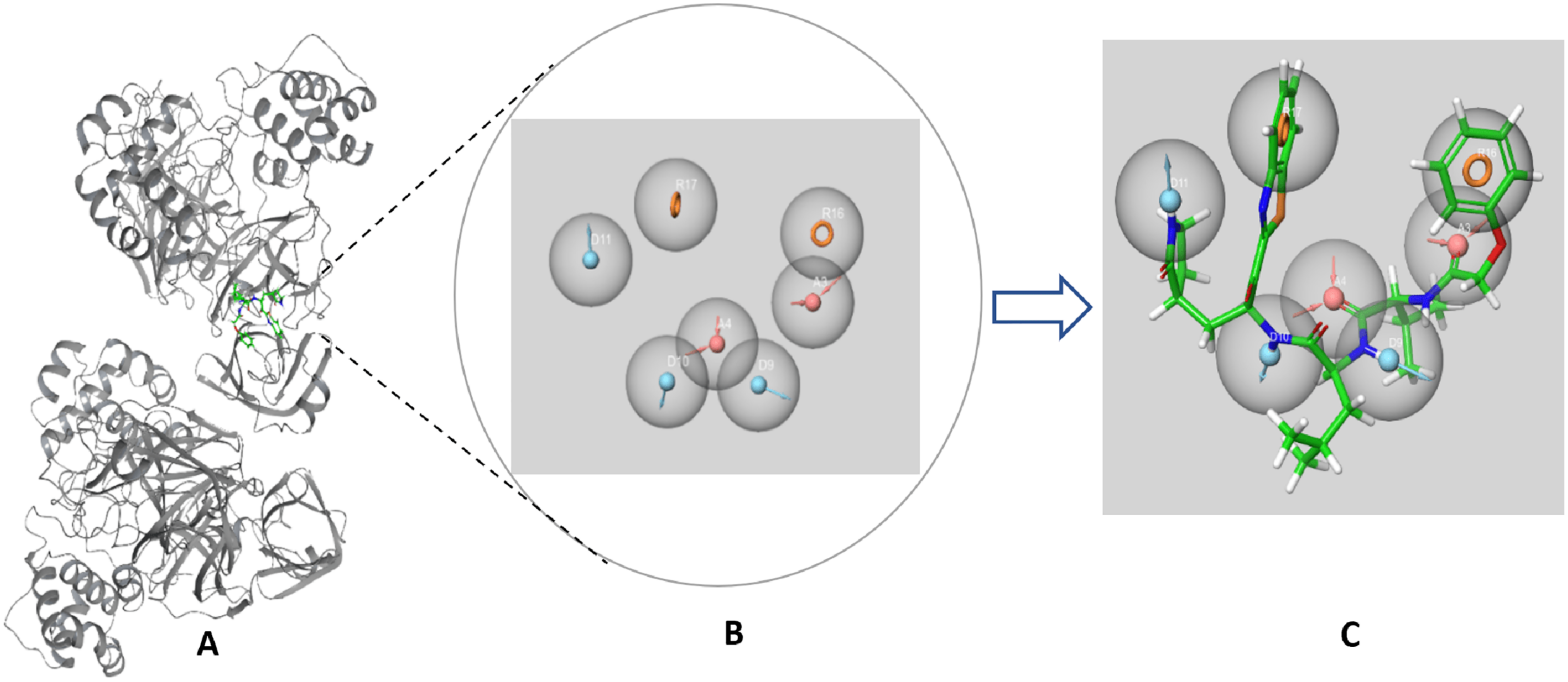
The generated e-pharmacophore hypothesis based on the binding of compound III (K_i_ = 4.1 nM) which exhibited the highest binding affinity to the protein. (A) 3D model of the crystal structure of SARS-CoV-2 3CL protease (PDB ID: 6M2N) complexed with the chosen standard compound **III**; (B) the generated hypothesis feature set (AADDDRR); (C) compound **III** overlayed on the top of the hypothesis.

The SARS-CoV-2 M^pro^ protein structure employed in this study (PDB: 6M2N) included 88.2% residues in the most favorable area and 10.9% residues in the additional allowed regions (Fig. S1). Compound III was used to generate a ligand-protein-complex-based hypothesis. The compound was chosen for its reported potent activity against SARS-COV-2 M^pro^, with an inhibitor constant (K_i_) of 4.1 nM and an inhibitory concentration (IC_50_) of 1.7 μM (Gao et al., 2021; Tian et al., 2021). Compound **III** violation of Lipinski’s rule of five meant (due to its high molecular weight of 635) meant that it is unable to be used for oral drugs development. However, its potent activity presented the compound as an excellent control to use in identifying new SARS-COV-2 M^pro^ with better pharmacokinetic properties. As illustrated in Fig. 2, seven pharmacophore sites were predicted, and the final hypothesis consisted of two-aromatic rings (R16 and 17), two H-bond acceptor sites (A3 and 4), and three hydrogen bond donor sites (D9, 10 and 11).

The generated hypotheses were validated based on the difference of score between the active and non-active compounds. Non-active compounds (decoys) were acquired employing DUD-E online service. Out of the two generated hypothesis the ligand-protein-complex-based hypothesis demonstrated adequate sensitivity and specificity through exhibiting a receiver operating characteristic (ROC) value of 0.83 and area under the accumulated curve (AUAC) of 0.85 (Table 1). Due to the poor specificity of the structure-based hypothesis, with ROC value of 0.53 and AUAC value of 0.58 (Table 1), it was deemed unfit for screening a large library. The graphical representation for the validation of the ligand-protein-complex-based hypothesis is illustrated in Fig. S2.

**Table 1:** The generated pharmacophores and their validation.

Pharmacophore characteristics	Structure-based hypothesis	Ligand complex-based hypothesis
Total Features	5	7
Feature Set*	AADDD	AADDDRR
ROC	0.53	0.83
AUAC**	0.58	0.85

**Notes:**

* A—hydrogen bond acceptor feature, D—hydrogen bond donor feature, H—hydrophobic feature, N—negative ionizable feature, S—shape feature.

** AUAC—area under area under an accumulation curve.

### Phase screening

Using the second generated hypothesis, Phase screening of the prepared library’s 20 million compounds was performed. Phase screening matches the structures of the tested compounds to the generated 3D e-pharmacophore. Like the restrictions imposed on the test set, a compound had to match five of the seven pharmacophoric sites to be considered active. The Phase screening identified 6,384 hits as positive match out of the tested 20 million screened compounds. Given that the phase screening does not consider the affinity of the screened compounds to the active site, further experiments were required to rank the affinity of the recognized hit compounds to the SARS-COV-2 M^pro^.

### Structural based virtual screening (SBVS)

SBVS was utilized to rank the 6,384 compounds generated from the phase screening based on their binding affinity to the active site. Docking scores, GlideScores, ligand efficiencies, and lipophilic and hydrogen bonding interactions were among the metrics studied. A further molecular modeling investigation was conducted for the top 30 hits identified from the SBVS (Table S2) since the SBVS approach can assess a large volume of molecules in a short amount of time, however the speed sometimes comes at the expense of accuracy leading to potential false positives.

### Molecular docking study

The results of docking the top 15 hits are displayed in Table 2.

**Table 2:** Docking scores and 2D structures of the top 15 hits from the molecular docking study.

Hit	Code	Structure	No. of sites matched	GlideScore (Kcal/Mol)
**1**	ZINC000000639429	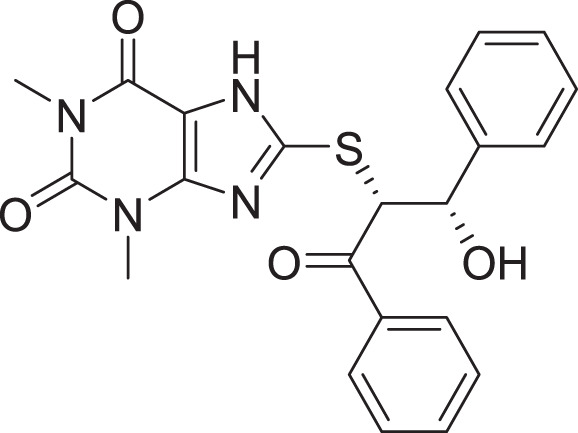	5	−8.706
**2**	ZINC000000996474	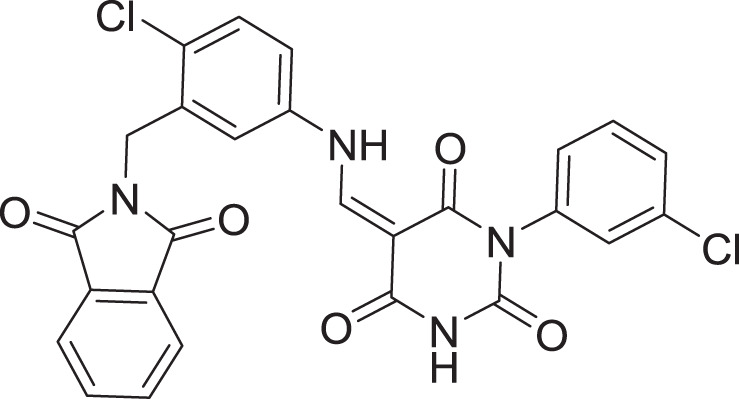	5	−8.493
**3**	ZINC000000788680	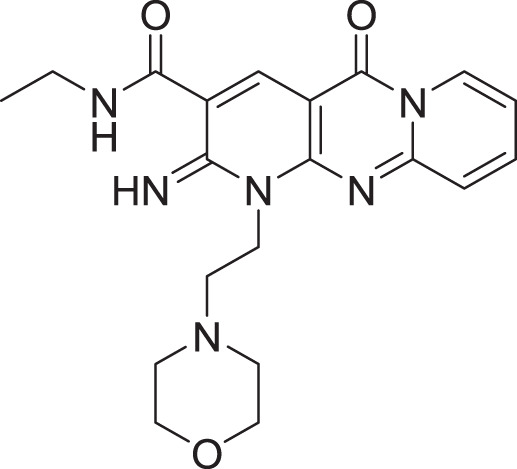	5	−8.49
**4**	ZINC000000727953	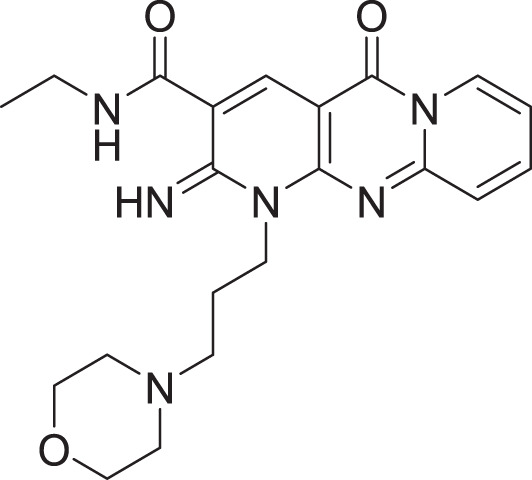	5	−8.451
**5**	ZINC000000865538	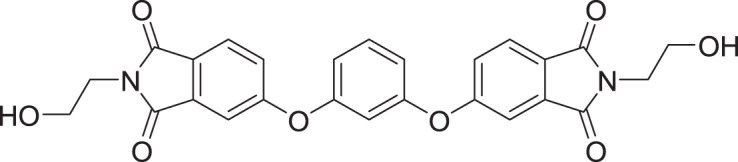	5	−8.416
**6**	ZINC000000788703	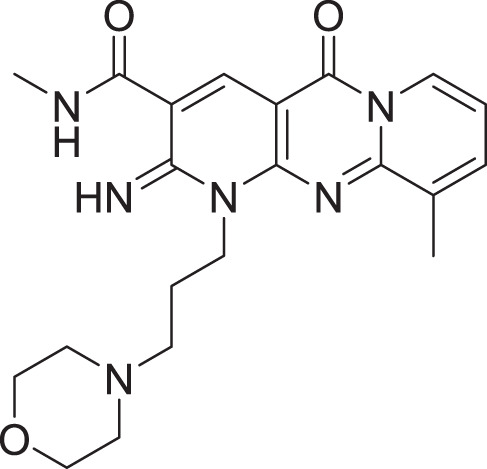	5	−8.349
**7**	ZINC000000788677	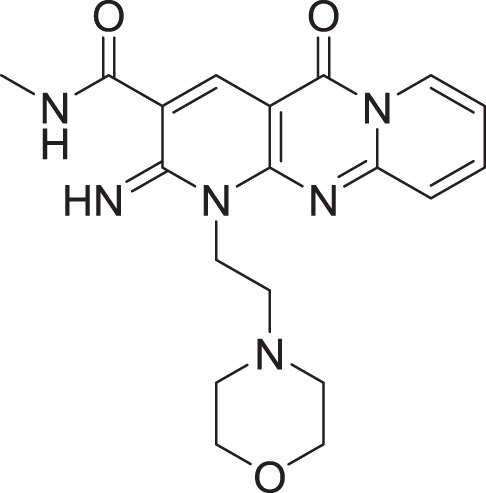	5	−8.309
**8**	ZINC000000996257	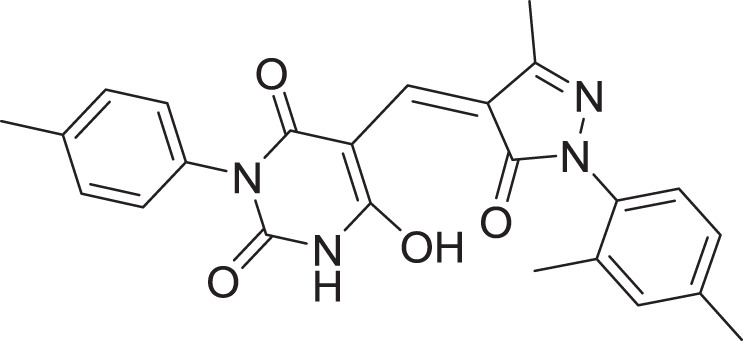	5	−8.265
**9**	ZINC000000727950	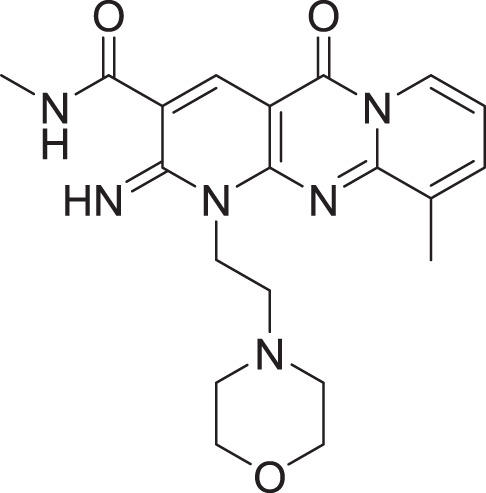	5	−8.236
**10**	ZINC000000645906	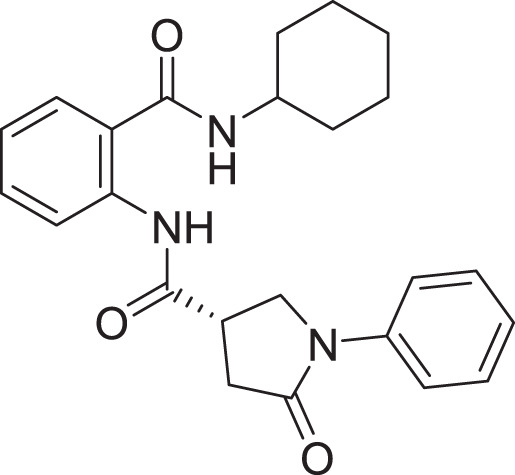	5	−8.213
**11**	ZINC000000043974	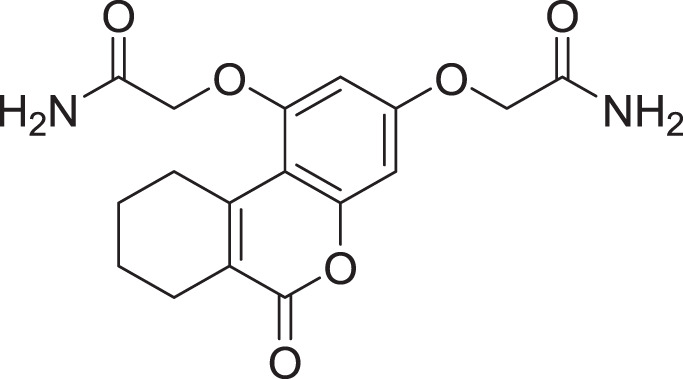	5	−8.187
**12**	ZINC000000688914	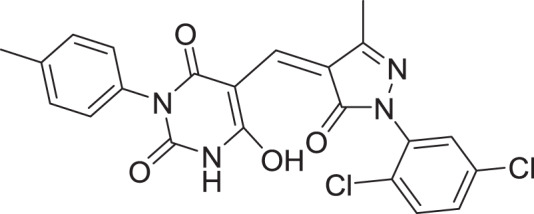	5	−8.179
**13**	ZINC000000728386	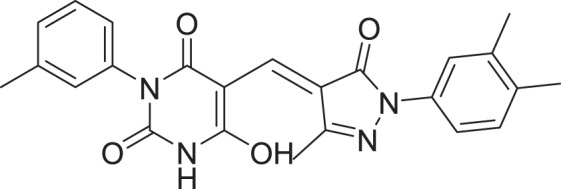	5	−8.177
**14**	ZINC000000626028	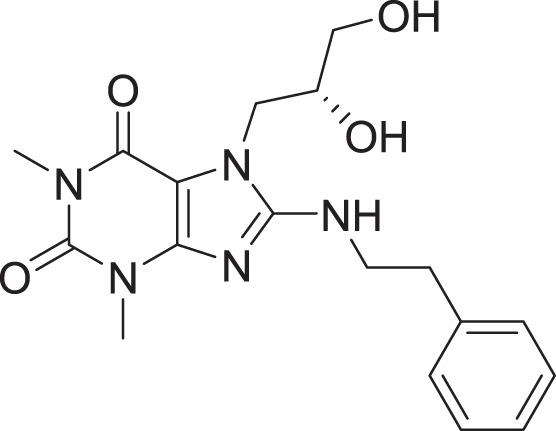	5	−8.169
**15**	ZINC000000936880	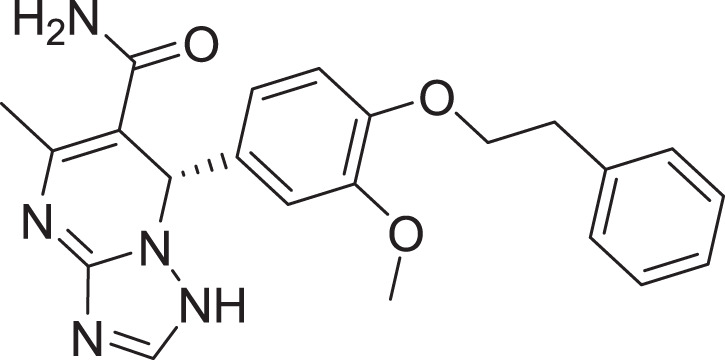	5	−8.132
**16**	Standard compound (**III**)	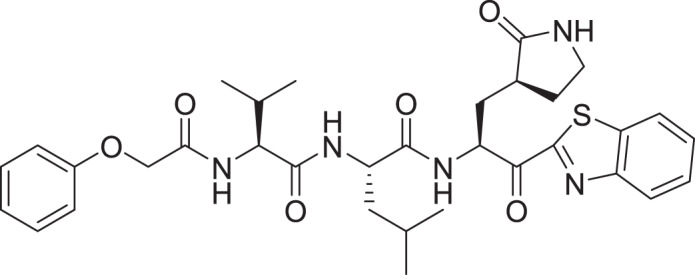	5	−8.127

**Figure 3:**
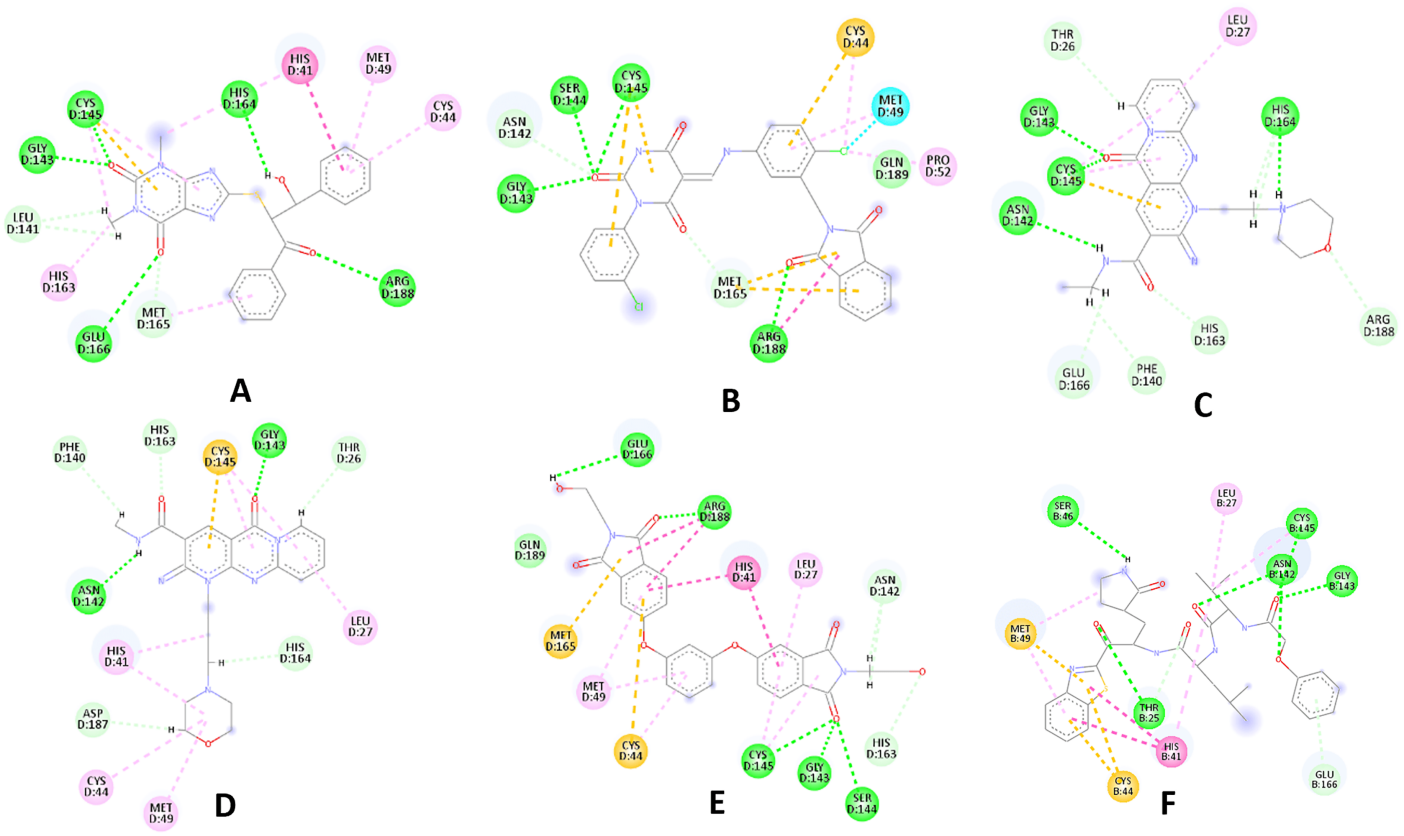
2D interaction patterns of the hit compounds 1 (A), 2 (B), 3 (C), 4 (D), 5 (E) and the standard III (F) in complex with SARS-CoV-2 Mpro (3CLpro). Favorable interactions are color coded as follows: green—hydrogen bonds, yellow–π–sulfur, orange–π–charge, deep pink–π–π stacking interactions, purple–π–sigma interactions, light pink–hydrophobic interactions, light green–carbon–hydrogen interaction, red–unfavorable interaction.

Among the top hits, compounds **3**, **4**, **6**, **7** and **9** (GlideScores of −8.49, −8.45, −8.35, −8.30, and −8.23 kcal/mol, respectively) shared high chemical structural similarity exhibiting a comparable strong pattern of interaction with main protease, which indicates a valid docking approach. Similarly, hits **12** and **13** were structurally similar, highlighting the ability of the generated e-pharmacophore in identifying structurally similar hits. The 2D interaction patterns of top six hits **1**‒**5** and the standard (**III**) are illustrated in Fig. 3. The 2D interaction patterns of the remaining hits are demonstrated in Figs. S3–S12.

**Figure 4:**
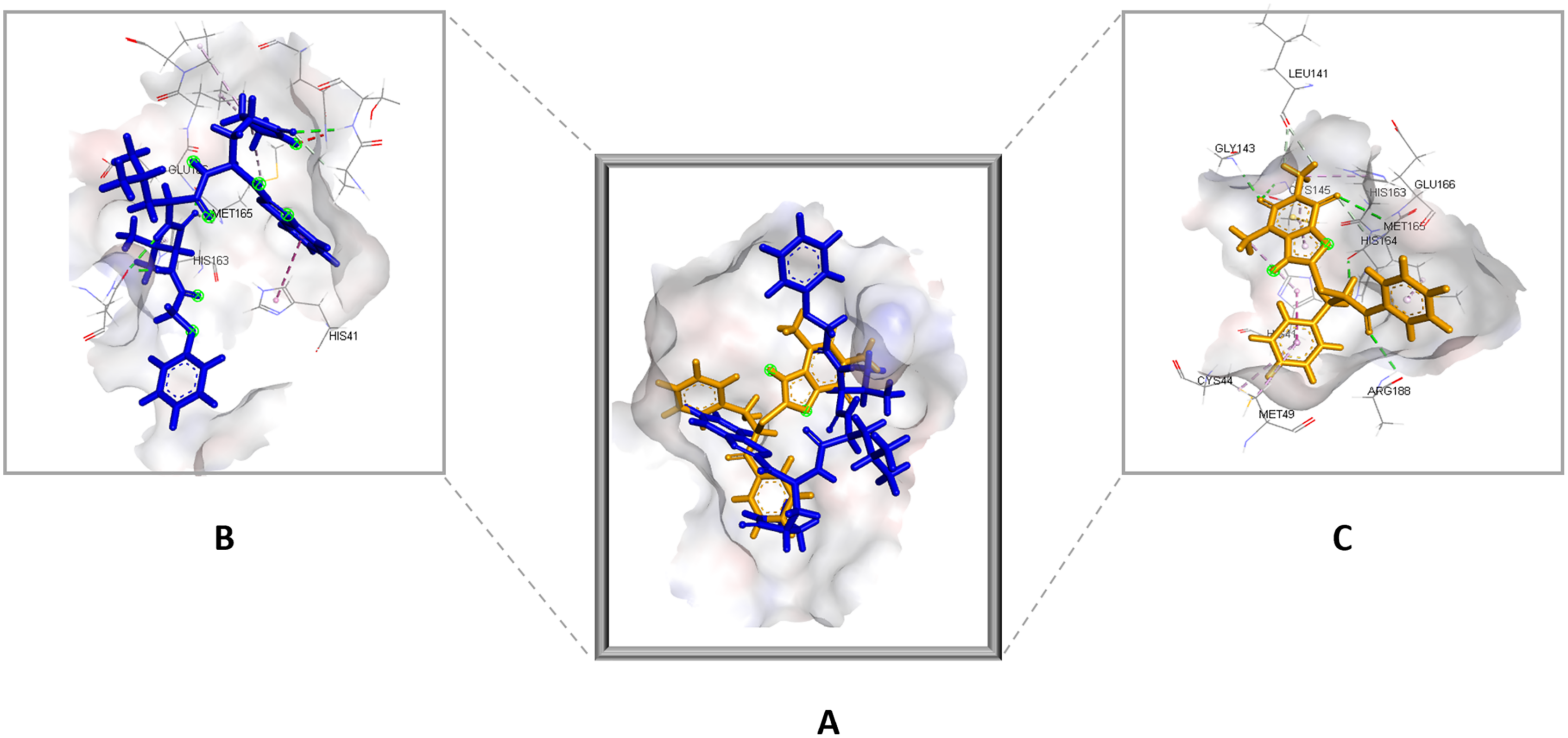
(A) The top hit compound 1 (orange) overlayed on top of the standard compound III (blue) inside the binding cavity of M^pro^. (B) and (C) illustrate the 3D docking poses of the standard (blue) and compound **1** (orange), respectively.

Compared to the standard (**III**), the top 15 hit compounds exhibited promising binding interactions when complexed with SARS-CoV-2 M^pro^. Due to the differences of size and chemical structure between compound **1**, (8-(((1S)-1-hydroxy-3-oxo-1,3-diphenylpropan-2-yl)thio)-1,3-dimethyl-3,7-dihydro-1H-purine-2,6-dione), and the standard (**III**), the smaller hit (**1**) displayed a better fit inside the binding cavity due since it was able to form a higher number of favorable interactions (Fig. 4). The top hit (**1**) established four hydrogen bonds with the active site; three of them were formed between the carbonyl groups of its purine moiety and GLY143 and CYS145 residues of the target protein. Another hydrogen bond was established between the hydroxy group and HIS164 residue. The presence of several hydrogen bonds along with its favorable orientation inside the binding cavity of the SARS-COV-2 M^pro^ predicts its validity as a potential potent inhibitor.

The remaining top hits displayed similar binding modes to compound **1**, with the majority retaining the hydrogen bonds with GLY143 and CYS145 residues. Interestingly, even though compounds **3**, **4**, **6**, **7** and **9** possessed high structural similarity, the length of the “linker” moiety altered their binding modes significantly, with compound **6** (Fig. S3) displaying the lowest number of favorable interactions.

### Pre-dynamic MM-GBSA calculations

MM-GBSA calculations were carried out for the top 15 hits and the standard (**III**). The top hit (**1**) displayed the highest free energy (−130.82 kcal/mol), predicting the stability of its complex with the Mpro and validating the docking results. The remaining hits displayed comparable free energy when compared to the standard (−82.14 kcal/mol), with only compound **14** exhibiting a significantly lowered free energy of −66.85 kcal/mol (Table 3).

**Table 3:** MM-GBSA calculations for compounds 1–15 and III.

Cpd	MM-GBSA (kcal/mol)
**1**	−130.82
**2**	−87.47
**3**	−85.29
**4**	−78.93
**5**	−88.32
**6**	−81.39
**7**	−78.35
**8**	−79.57
**9**	−79.43
**10**	−79.08
**11**	−76.27
**12**	−78.91
**13**	−75.14
**14**	−66.85
**15**	−73.80
Compound **III**	−82.14

### Molecular dynamics (MD) study

#### Stability analysis of ligand–protein complexes (RMSD calculations)

**Figure 5:**
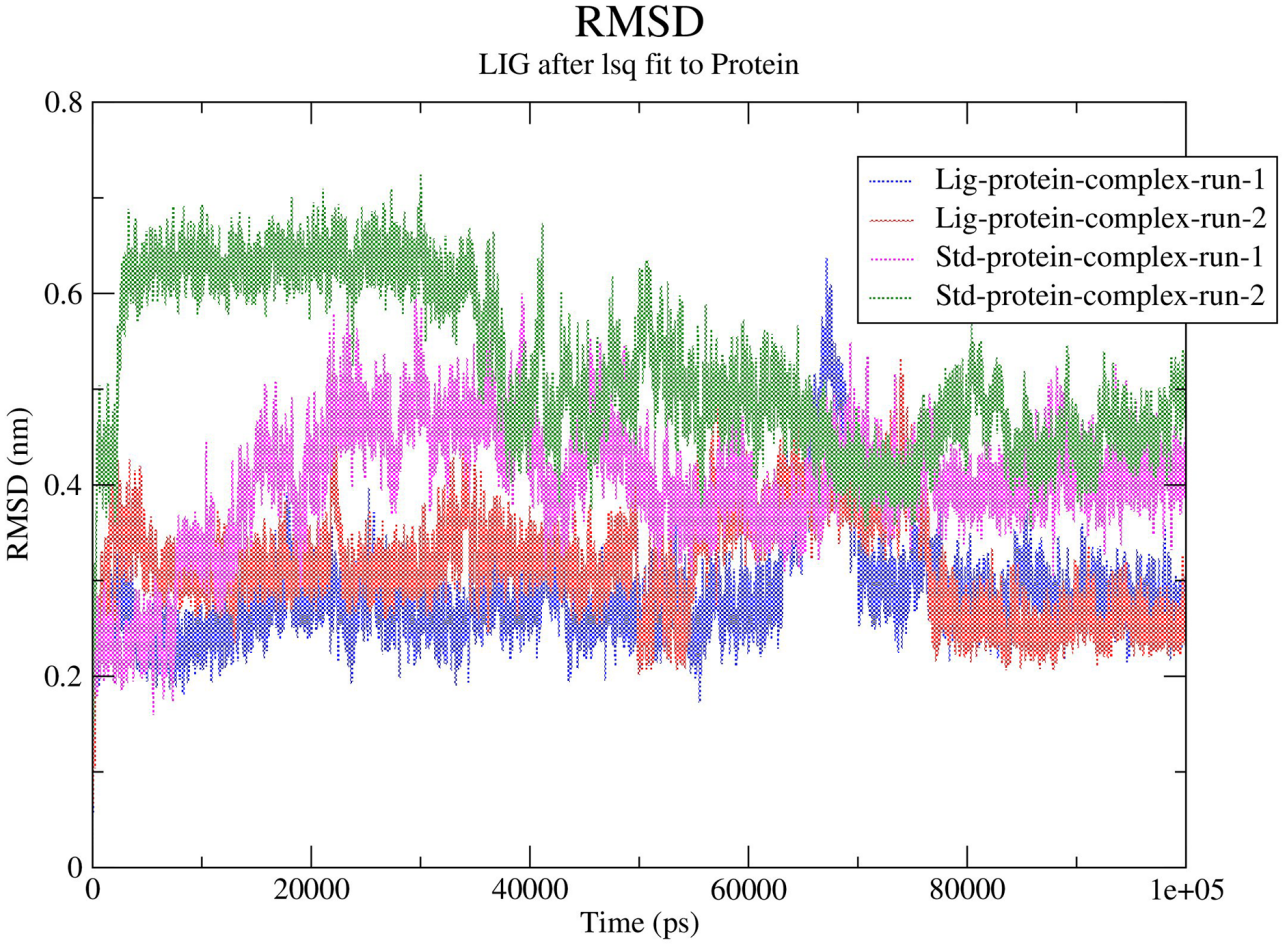
RMSD calculations.

The RMSD values for the protein Cα atoms, compound **1**, and the standard **III** were calculated by aligning the MD production phase trajectories with their initial structures in three simulation experiments. The complexes of compound **1** and SARS-COV-2 M^pro^ exhibited average RMSD values of 2.8 and 3.1 Å in the first and second MD runs, respectively. The complexes of compound **1** with the SARS-COV-2 M^pro^ during both MD runs showed stability after the initial 10 ns of the simulation. However, during both MD runs the RMSD of compound **1**-SARS-COV-2 M^pro^ complexes showed sudden fluctuation for a duration of 10 ns between 65 and 75 ns which quickly stabilized again to the initial RMSD. Conversely, the standard compound- SARS-COV-2 M^pro^ complexes showed average RMSD of 4.0 and 5.2 Å, in the first and second MD runs, respectively. In both runs the standard-SARS-COV-2 M^pro^ complexes required around 50 ns to reach stability. The changes in the RMSD values of compound **1** (referred to as Lig) and the standard compound (**III**, referred to as Std) during both MD runs is illustrated in Fig. 5.

In order to predict the ligand fluctuation inside the binding cavity throughout the simulation, the RMSD for each ligand was also calculated. Compound **1** RMSD was very stable in both MD run with an avearge RMSD value of 1.9 Å. On the other hand, the standard compound (**III**) required 40 ns to reach a stable conformation, exhbiting average RMSD values of 2.5 and 2.9 Å in the first and second MD simulations, respectively. The full MD simulation of both runs of the standard (**III**) and compound **1** can be viewed in Videos 1–4, while the changes in the RMSD of compounds **1** and **III** is represented in Fig. S13.

#### RMSF calculations

**Figure 6:**
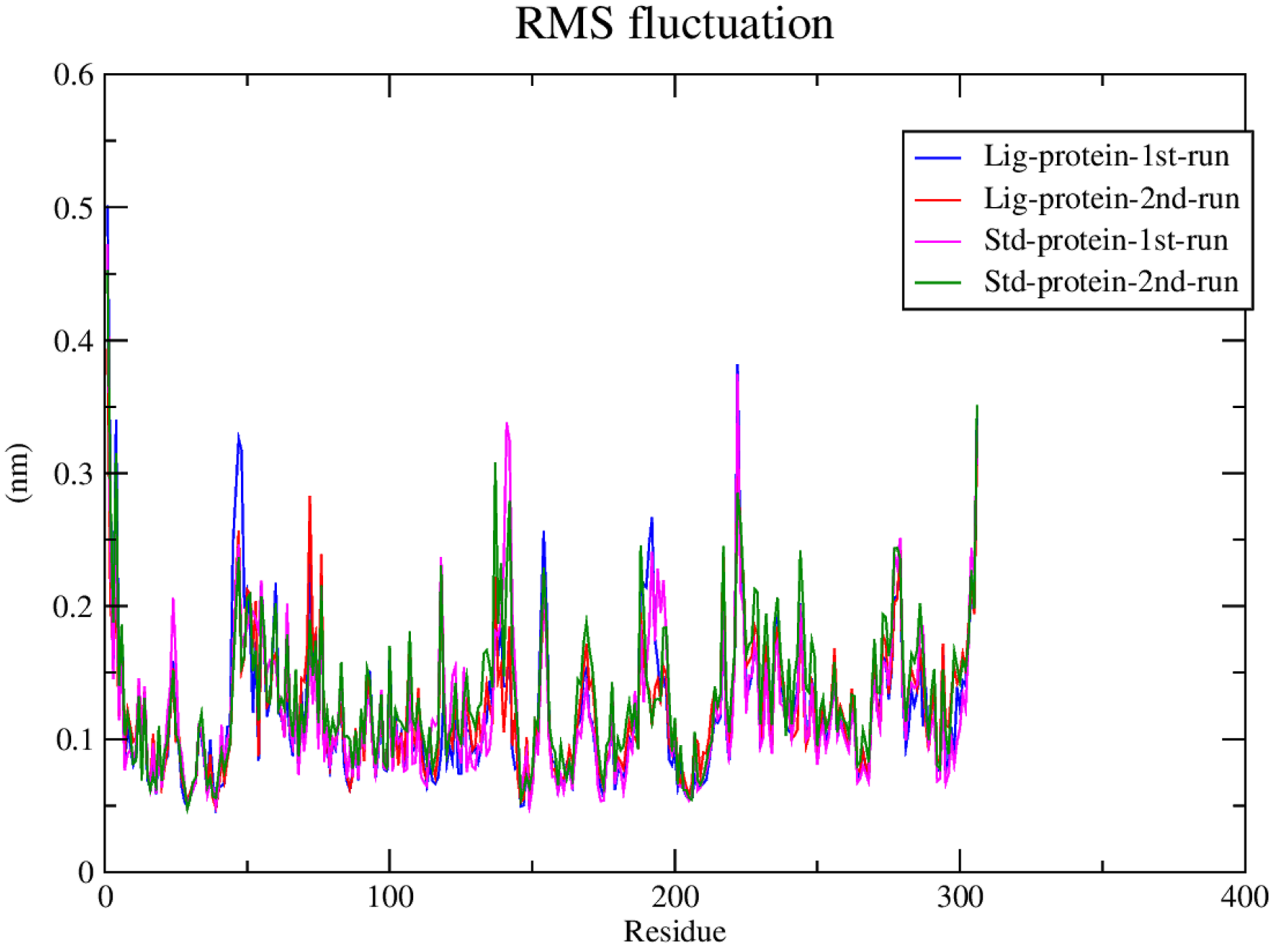
RMSF of compound 1 and the standard-protein complexes.

Subsequently, the alpha carbons RMSF (root-mean-square fluctuation) was calculated to evaluate the structural mobility and flexibility. The RMSF analysis showed comparable stability and flexibility between the standard (**III**) and compound **1** throughout all four molecular dynamic simulations as exhibited in Fig. 6.

#### Hydrogen bonds

**Figure 7:**
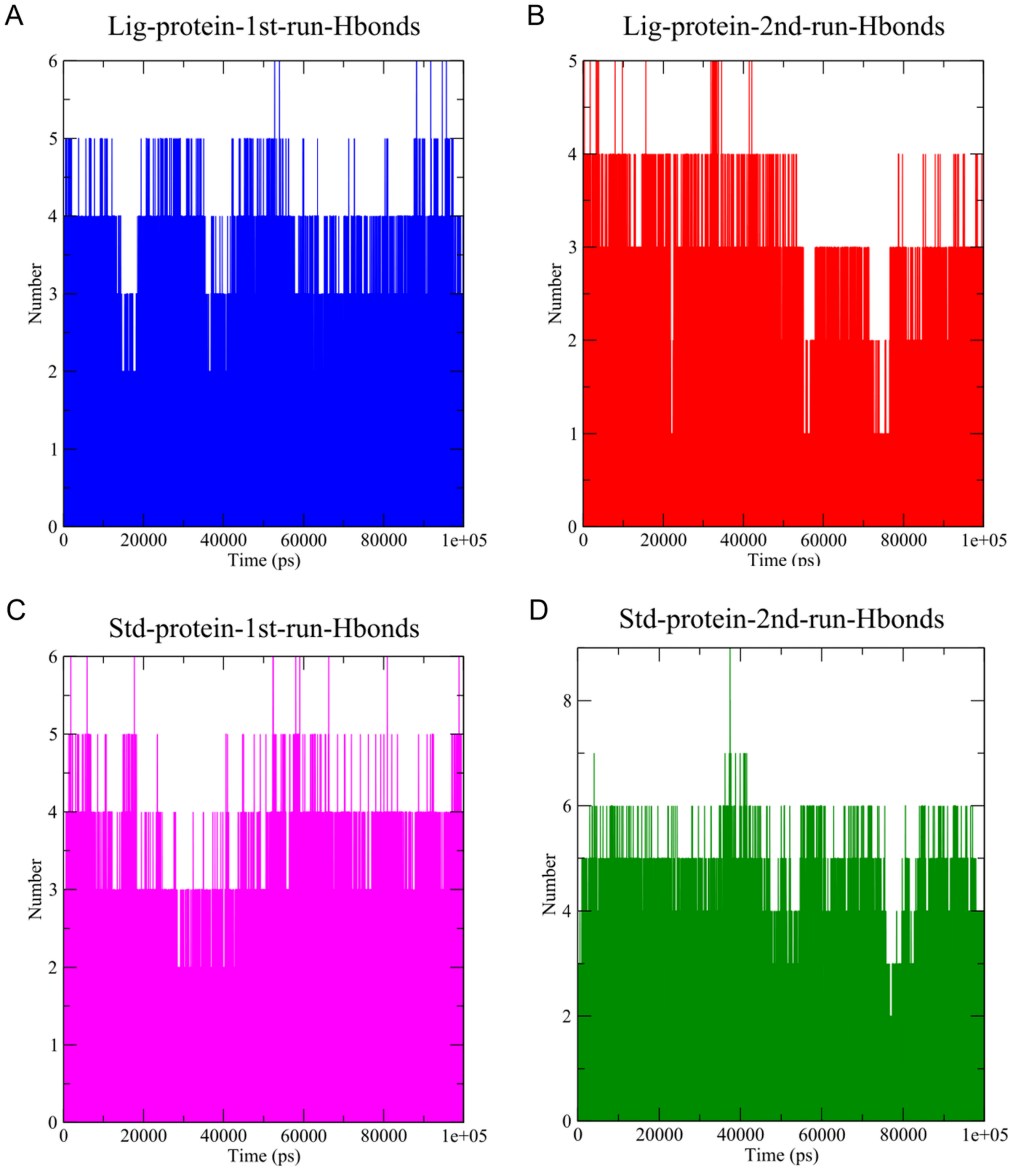
The number of hydrogen bonds established. (A) Hydrogen bonds established by compound 1-protein complex in the 1^st^ run**. **(B) Hydrogen bonds established by compound 1-protein complex in the 2^nd^ run. (C) Hydrogen bonds established by standard-protein complex in the 1^st^ run**. **(D) Hydrogen bonds established by standard-protein complex in the 2^nd^ run.

The stability of MD simulation was evaluated by measurement of the intermolecular H-bond(s) developed between the SARS-COV-2 M^pro^ complexes of compounds **1** and the standard **III**. The complexes of compounds **1** and **III** with the protease maintained two hydrogen bonds throughout the whole 100 ns simulation (Fig. 7). Furthermore, both complexes maintained four hydrogen bonds throughout the majority of the simulations. All complexes established hydrogen bonds with an average donor-acceptor distance of 2.75 Å (Fig. S14).

**Figure 8:**
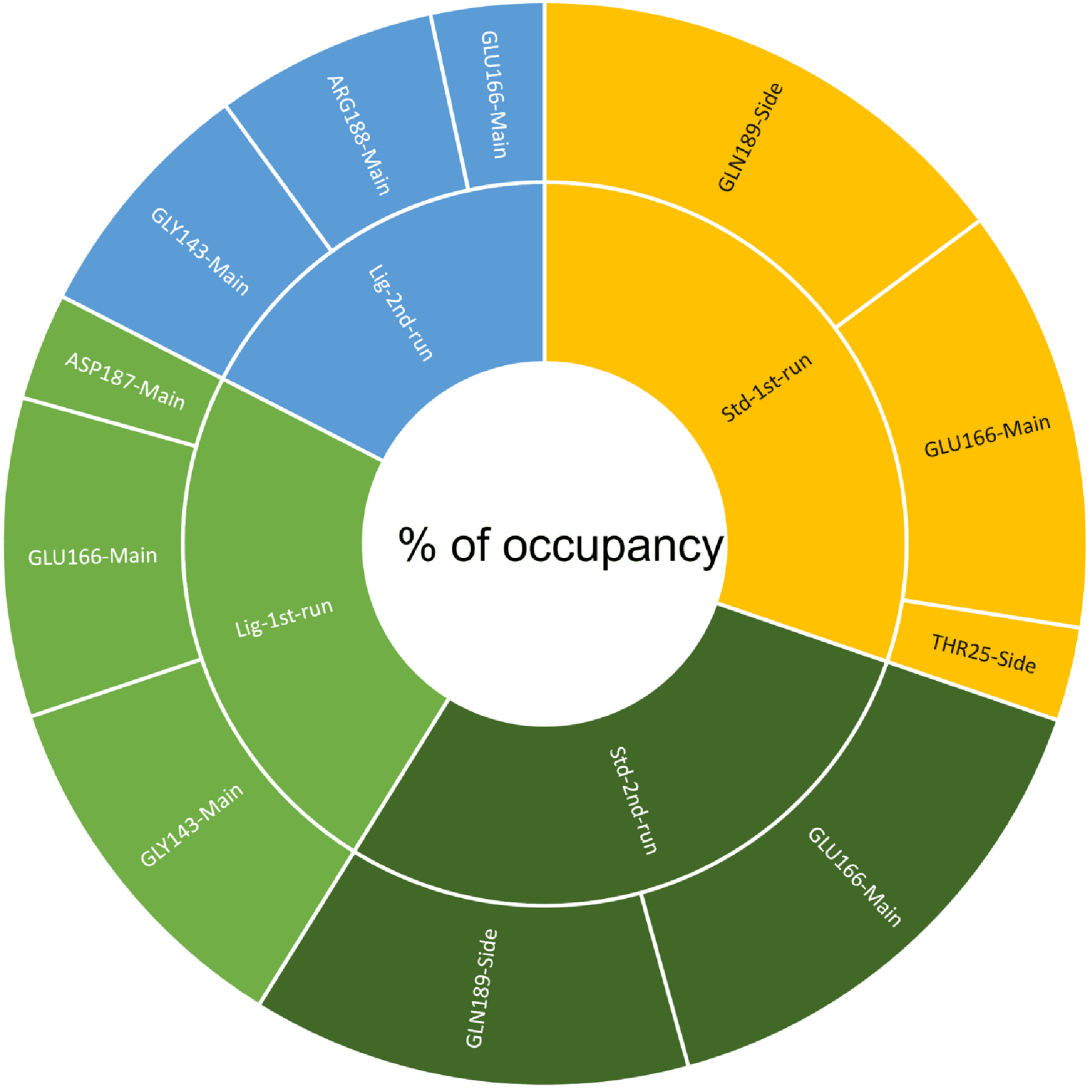
The percentage of occupancy each residue participating in H-bonding.

A cut-off criterion comprising of a distance ≤3.0 Å and an angle of ≥120 degree between the proton donor and acceptor atoms, was used to calculate the hydrogen bond (H-bond) occupancy value as a percentage (Fig. 8). Among the hydrogen bonds established by compound **1** during the first run, GLY143 of the main chain established the strongest hydrogen bond populating 45.52% (Fig. 8). Meanwhile, GLU166, and ASP187 amino acid residues contributed to 39.31% and 13.21%, respectively. Similarly, during the second molecular dynamic simulation of compound **1**, GLY143 residue contributed to the most occupancy (30.76%), while GLU166 AND ARG188 contributed to 13.99% and 27.41% of the established hydrogen bonds, respectively. Conversly, hydrogen bonds with GLU166 of the main chain and GLN189 contributed to the majority of hydrogen bonds of the standard compound in both MD smilations (Fig. 8).

**Figure 9:**
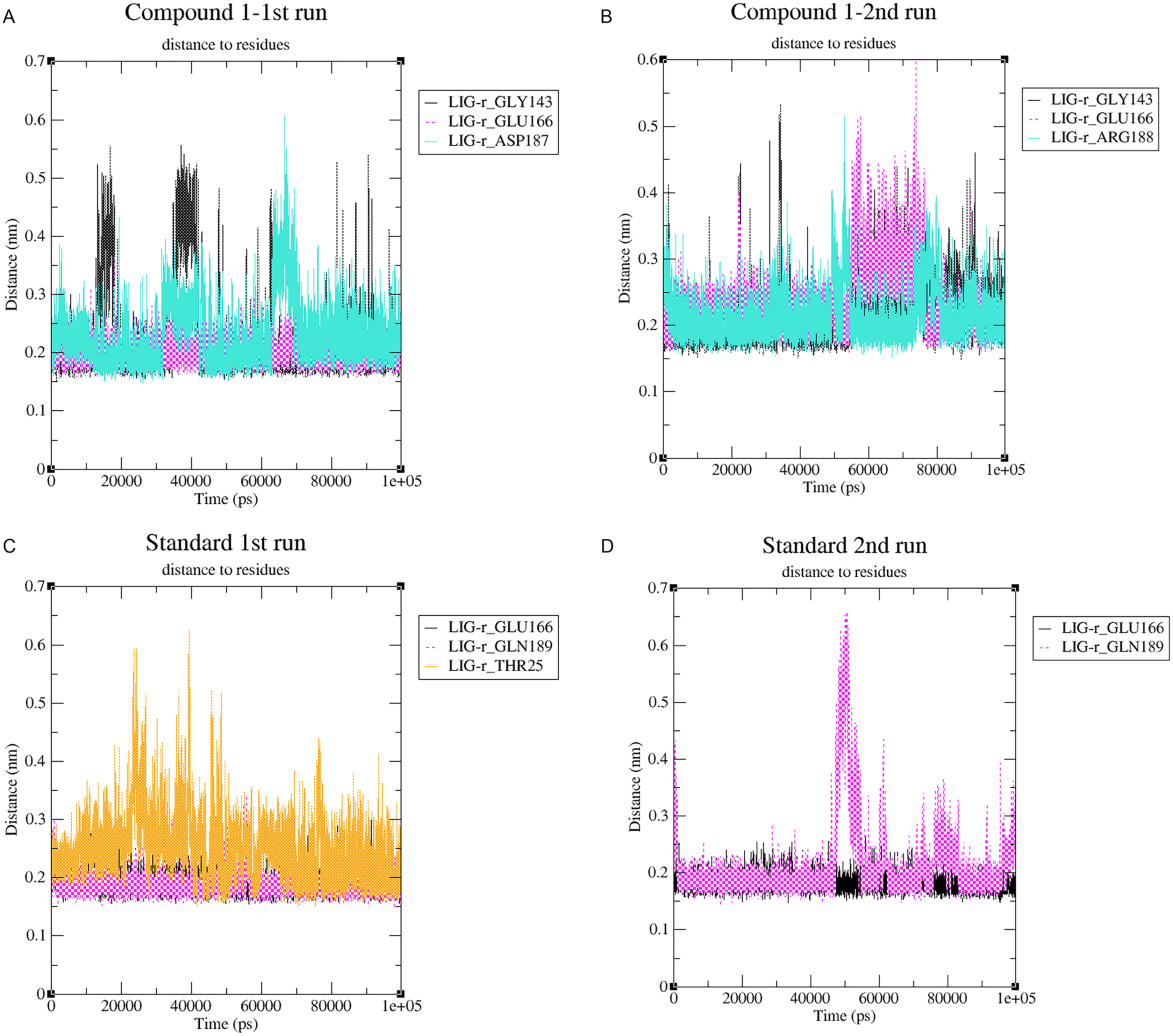
(A–D) The changes in distance between key binding-site amino acids and the ligands over time during each MD simulation.

Changes in the distance of the compounds to the amino acid residues contributing to substantial hydrogen bonds, were assessed by studying the variation of distance between Cα atoms of the binding cavity and the compounds throughout the MD simulations. In all simulations the average distance between the compounds and the hydrogen bond contributing residues was below 2.5 Å (Fig. 9). During the MD simulations of the compound **1** the distance, between the compound and both GLY143 and GLU166 amino acid residues, showed brief fluctuations (Figs. 9A and 9B). The distance of the standard to GLU166 throughout both MD simulations did not exhibit any fluctuation. However, during both MD simulations of the standard, there was a marked fluctuation in the distance of the standard to GLN189 and THR25 amino acids (Figs. 9A and 9B).

#### Radius of gyration (Rg) and SASA

As illustrated in Fig. S15, both complexes (compound **1**- and **III**- protein complexes) during the two runs displayed a stable curve with a decrease in acceleration from 2.5 nm to a plateau at below 2.25 nm by the end of the 100 ns simulation, indicating a stable ligand-protein complex simulation and an appropriate simulation time (Fig. S15). SASA is a parameter computed using GROMACS’s “gmx mpi sasa” module that accounts for the solvent-accessible surface area (SASA). Both compound **1** and the standard compound (**III**)-SAR-COV-2 M^pro^ complexes exhibited an average SASA of ~150 nm^2^ during both runs of each complex (Figs. S16 and S17).

#### PCA and FEL

**Figure 10:**
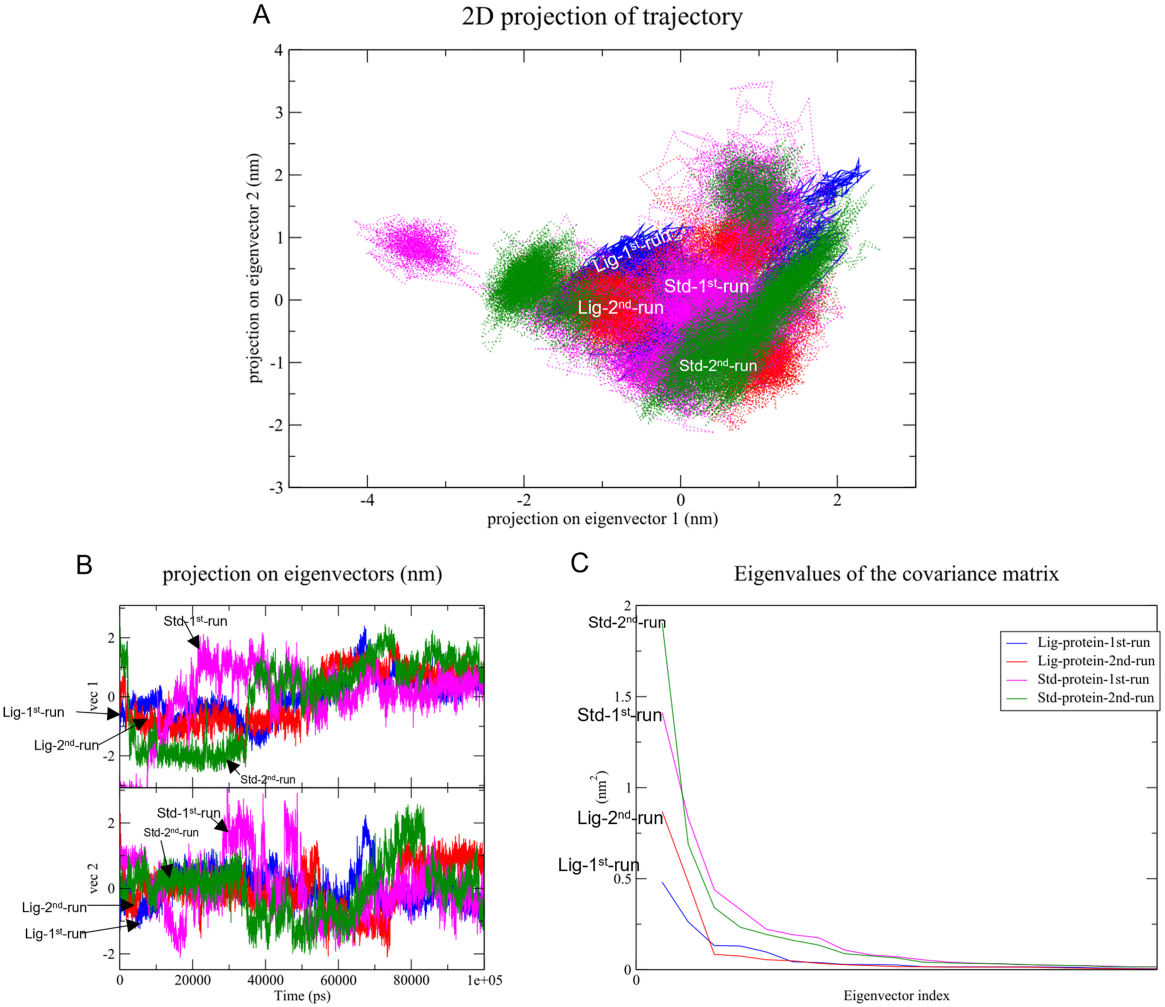
Principal component analysis. (A) Conformational projections of SAR-COV-2 M^pro^depicted as2D projections of trajectories on eigenvectors (EVs). (B) Trajectory projections for both EVs with respect to time. (C) Eigenvalues plotted against eigenvector index (only the first 20 eigenvectors were included)

Changes in the correlated motions throughout the MD simulation were assessed through PCA or essential dynamics analysis. Figure 10A depicts the conformational sampling of SAR-COV-2 M^pro^ along with its docked complexes in the essential subspace. Figures. 10A and 10B portray the structural conformations of SAR-COV-2 M^pro^ and the eigenvector (EV)-1 and EV-2 projected by C^α^ atoms. Significant overlapping of the stable clusters of the studied complexes was observed (Fig. 10A). Additionally, both compound **1** and the standard-protein complexes occupied the same conformational space during both runs (Fig. 10B). However, EV1 exhibited an increased dynamic when compared to EV2 (Fig. 10B). The first 10 eigenvectors accounted for ~80% of the fluctuations demonstrated by the studied complexes (Fig. 10C). Figure 10C illustrates that compound **1** (referred to as Lig) in both simulations exhibited lower correlated motions with the residues when compared to the standard compound.

PC1 and PC2 were used to calculate and plot the FEL diagrams to verify the spatial positions of atoms in a system. In the FEL lower energy is indicated by a free energy landscape with a deeper shade of blue. The FEL figures illustrate the free energy landscapes projected onto the first two principal components of the standard compound (**III**) and compound **1** complexes for backbone atoms of SAR-COV-2 Mpro during the two runs of 100 ns MD simulations. Both the standard compound (**III,**) and compound **1** exhibited comparable free energy surfaces, possessing multiple energy basins which indicate the presence of more than one stable conformation. However, compound **1** energy basins were more conical while the standard energy basins in both runs were flatter.

#### MM-PBSA free energy calculations

MM-PBSA calculations were conducted for all four complexes with the results presented in Table 4. Generally, the free energies of compound **1**-SARS COV-2 M^pro^ complexes were very similar in the two MD dynamic simulations, exhibiting average binding free energies of −126.77 and −120.77 kJ/mol in the first and second MD runs, respectively. Although, the standard displayed a higher average binding free energies than compound **1** in both MD simulations, there was a high difference between the free binding energies displayed by the standard complexes during the first and second MD simulation with total free binding energies of −167.49 and −204.20 kJ/mol, respectively. To explain the MM-PBSA results, the Van der Waals (hydrophobic, LJ-SR) interaction energies of SARS COV-2 M^pro^-compound **1** and the standard (**III**) complexes were calculated and plotted in Figs. S18A and S18B.

**Table 4:** MMPBSA calculations.

Complex	ΔE binding (kj/mol)	ΔE Electrostatic (kj/mol)	ΔE Vander Waal (kj/mol)	ΔE polar solvation (kj/mol)	SASA (kJ/mol)
**1-first run**	−126.77 ± 16.77	−83.17 ± 19.58	−183.66 ± 11.31	160.14 ± 11.09	−20.08 ± 0.78
**1-second run**	−120.77 ± 14.44	−77.46 ± 16.76	−191.00 ± 11.00	168.63 ± 13.02	−20.21 ± 0.99
**III-first run**	−167.49 ± 15.09	−121.48 ± 12.32	−198.82 ± 12.89	176.91 ± 13.02	−24.10 ± 1.58
**III-second run**	−204.20 ± 14.44	−159.21 ± 18.92	−269.47 ± 12.15	252.24 ± 115.11	−27.76 ± 1.13

### *In silico* pharmacokinetics study

As illustrated in Table 5, ADME (absorption, distribution, metabolism, and excretion) profiles of the standard and the top hit were examined to predict their pharmacokinetic properties using the Swiss ADME server. The two Lipinski violations of the standard stemming from its high molecular weight and log P (partition coefficient) predict it to possess poor absorption by the gastrointestinal system which indicates it oral unavailability. Compound **1**, on the other hand, did not violate any of the Lipinski’s rule of 5. In addition, it possesses a lower molecular weight than the standard and a higher water solubility. Furthermore, compound **1** exhibited a topological polar surface area (TPSA) of 135.28, suggesting a good gastrointestinal absorption. Accordingly, compound **1** is predicted to be a promising lead for developing oral SARS-COV-2 M^pro^ inhibitors.

**Table 5:** The pharmacokinetic profile of compound **III** **and the top predicted hit** **1**.

Cpd	Mwt	TPSA	LogP	Solubility	BBB permeability	GIT absorption	Lipinski rule violations
**III**	635.77	183.83	5.02	Poor	No	Low	2
**1**	436.48	135.28	2.75	Moderate	No	High	0

## Discussion

In an attempt to identify SARS-COV-2 M^pro^ inhibitors, a multi-step approach designed and conducted *in silico*. The first step in this process was to ensure the validity of the employed crystal structure. This was carried out *via* checking the generated Ramachandran plot, which predicted that more than 99% of the amino acid residues were within the allowed regions. The next step involved generation of validated hypotheses to screen a large database, composed of 20 million compounds, for SARS-CoV-2 M^pro^ inhibitors based on predicted pharmacophoric features.

Tian et al. (2021) recently published a review gathering the identified small molecules inhibitors for COVID-19 and their different modes of actions. Among the reported molecules, 30 small molecule inhibitors have a potent *in vitro* inhibitory activity against a SARS-CoV-2 M^pro^, which were used herein as the test set (Table S1) to develop two different hypotheses arising from two different approaches. The first approach utilized the test structures to generate a five-point hypothesis using Maestro’s generate structure-based hypothesis module. The second hypothesis was generated *via* docking the test set into the binding cavity of SARS-COV-2 M^pro^ and using the binding pattern of the top hit, compound **III** (Table S1), to generate a hypothesis using the ‘Develop Pharmacophore from protein-ligand complex’ function of the Protein Preparation Wizard of the Schrodinger suite (Madhavi Sastry et al., 2013). Interestingly, the difference between the two hypotheses was the addition of a structure shape feature (aromatic rings) to the ligand complex-based hypotheses leading to an increase in the high specificity of the hypothesis.

Out of the two generated hypotheses, ligand-protein-complex-based hypothesis was selected to screen the prepared library of compounds as it exhibited a AUAC of 0.85 marking it as a valid hypothesis. Out of the 20 million compounds, only 6,384 compounds were predicted to enjoy at least five pharmacophoric features similar to those of the chosen hypothesis. SBVS was subsequently utilized to rank the 6,384 compounds based on their affinity to the binding cavity of the crystal structure.

The top 30 hits identified in the SBVS were subjected to a thorough molecular docking study to better understand their interaction profiles as well as weeding out any false positives from SBVS. The process of predicting the binding modes between a ligand and its intended target and correlating the resulting scores with the prospective activity are all useful applications of molecular docking in computer-aided drug design (CADD) studies (Gao, Yang & Zhu, 2010). Another advantage of molecular docking studies is their capacity to estimate the influence of certain amino acid mutations on the activity profile of the ligand (Vasina et al., 2022). Additionally, the visualization of the generated interactions from the docking study aids future improvement of the examined ligands to produce molecules with improved affinity properties (Umar et al., 2022).

Among the 30 compounds subjected to a molecular docking study, 15 compounds were predicted to possess superior binding affinity when compared to the standard compound (III, GlideScore = −8.12 Kcal/mol). The majority of the examined hits established a hydrogen bond with CYS145 increasing the confidence in their prospective as SARS-COV-2 M^pro^ inhibitors, as it was reported that potent inhibitors usually establish a hydrogen bond with the Cys145 residue (Tian et al., 2021).

Although molecular docking predicts the binding affinity, it fails to account for the effect of solvents on stability of the docked complexes (Wang, Lai & Wang, 2002). Consequently, the free energy estimated using the MM-GBSA method becomes an essential tool for validating the potential affinity of the docked complexes to their intended target (Slynko et al., 2014). The lower the calculated binding free energy of a ligand protein complex, the more stable it is predicted to be, as well as the higher its predicted activity and potency (Tripathi, Muttineni & Singh, 2013). The pre-dynamic MM-GBSA calculations predicted compound **1** to possess a considerably higher stability and biological activity when compared to the standard, warranting further investigation through a molecular dynamics study.

The function of viral proteases involves a broad range of structural flexibility, particularly at the active site amino acid residues (Bianchi & Pessi, 2002). Therefore, the top hit (**1**) and the standard (**III**) were subjected to molecular dynamic simulations (MDs) of 100 ns duration to test the stability of their ligand—protein complexes. Furthermore, MDs are regarded as particularly valuable tools for investigating the conformation space of ligand—target complex due to their higher efficiency when compared to other *in silico* tools such as molecular docking and mechanics energy minimization approaches which only perform static image analysis (Mortier et al., 2015). Validation of the molecular dynamics system was carried out by performing binding free energy calculations to assess the stability of the molecular dynamic systems.

In MD analysis, the root-mean-square deviation (RMSD) is employed to a assess the molecular deviation of a certain ligand compared to a defined reference structure, providing indication on the stability of the ligand–target stability and the validity of the adopted MD simulation protocol (Elhady et al., 2021). Except for a slight fluctuation during the period of 65 to 75 ns Compound **1** protein complexes experienced minimal fluctuations throughout both runs of the MD simulation indicating successful convergence on the target protein. Due to the flexible nature of the standard, especially highlighted when the methylphenoxyacetamide “tail” moiety of the standard changes its position after 40 ns of the MD simulation, the standard compound (**III**) required around 40 ns to reach stable conformation (Fig. S13). This fluctuation of the standard was reflected in the RMSD values of the standard-protein complexes in both runs which needed 40ns to reach stability. In conclusion, compound **1** during both MD simulations displayed a lower RMSD than the standard compound (**III**), indicating higher inhibitory potential when compared to the standard compound. Accordingly, the top hit is predicted to possess promising potential in inhibiting the M^pro^ of SARS-COV-2.

This conclusion is further validated after considering their low RMSF values which indicated the low flexibility of both complexes. The significance of the low RMSF values stems from the RMSF analysis being an essential tool in the identification of the rigid and flexible sections of the protein structure (Chiaramonte et al., 2018). RMSF is defined as the standard measure of deviation of a molecule from its initial position (Swetha, Ramaiah & Anbarasu, 2016). RMSF can be further applied to gauge the flexibility of the backbone residues of a protein structure along with any ligands involved (Klompas, Baker & Rhee, 2020).

Another tool which is useful in analyzing the stability of the complexes studied during the MD simulation is the number of hydrogen bonds established and maintained between the molecule and the binding site residues (Kumar et al., 2021). As both compound **1** and the standard were able to maintain at least three hydrogen bonds throughout the simulation which indicates that both molecules exhibit strong and stable binding affinity to the SARS-CoV-2 M^pro^. In all four molecular dynamic simulations GLU166 played a big part in the hydrogen bonds established between the compounds and the receptor, indicating its significance. Calculating the percentage of occupancy each residue involved in H-bonding with the studied compounds showed that both compounds **1** and the standard compound (**III**) established a strong bond with the GLU166 residue, indicating that the formation of a hydrogen bond with GLU166 is essential for the inhibitory activity. During the MD simulation the average distance between the compounds and the hydrogen bond contributing residues was below 2.5 Å which falls within the accepted range (McDonald & Thornton, 1994). These findings validate compound **1** as SARS-CoV-2 M^pro^ inhibitors. The increase in distance between compound **1** and GLU166 after 60 ns (Figs. 9A and 9B) explains the observed corresponding fluctuation the the compound’s RMSD (Fig. 5). This increase in the distace which affected both the RMSD and established hydrogen bonds of compound **1**, is due to the rotation of the compound around its own axis at 60 ns until it returns to its starting position leading to return of stabilization (Videos S1 and S2). The hydrogen bonds established by all complexes possessed an average donor-acceptor distance of 2.75 Å which is within the acceptable range indicating their validity.

The radius of gyration (Rg) is another useful implementation in understanding the folding properties and compactness of the protein–ligand complexes (Surti et al., 2020). Additionally, Rg can be utilized to predict the impact of a drug or a molecule on the protein structure and its ability to exert conformational changes (Sneha & George Priya Doss, 2016). The higher the Rg value, the more loosely packed the molecules inside a protein. On the other hand, lower Rg values indicate tight packing of the protein structure. In the case of a stable ligand-protein complex simulation, the radius of gyration would plateau on average (Lee et al., 2009). Both compound **1** and the standard (**III**) protein complexes displayed a decreasing acceleration plateauing at 2.5 nm, indicating a stable ligand-protein complex simulation and an appropriate simulation time. The SASA calculation can be used to predict the extent of conformational changes that occur during binding (Shahbaaz, Nkaule & Christoffels, 2019). Both complexes possessed comparable SASA averaging at ~150 nm^2^ in all four simulations. This result validates the reproducibility of the results and ensure the viability of the chosen study model.

In general, eigenvalues exhibits the magnitude of the motions through the direction while eigenvectors identify the direction of the motions (Gupta et al., 2021). Both compounds demonstrated the ability to maintain a strong hydrogen bond with the amino acid residues of SARS-CoV-2 M^pro^ binding cavity. 2D analysis of the projected motion of the protein in phase space along PC1 *vs* PC2 for both complexes showed that both ligand-protein complexes during both runs formed stable clusters. The presence of significant overlapping of the stable clusters in Fig. 10A indicates that the complexes occupied the same subspace. Which was further confirmed in Fig. 10B, where both compound **1** and the standard-protein complexes occupied the same conformational space during both runs (Fig. 10B) indicating the stability of the established complexes. Furthermore, compound **1** showed lower correlated movements when compared to the standard during both simulations (Fig. 10C).

**Figure 11:**
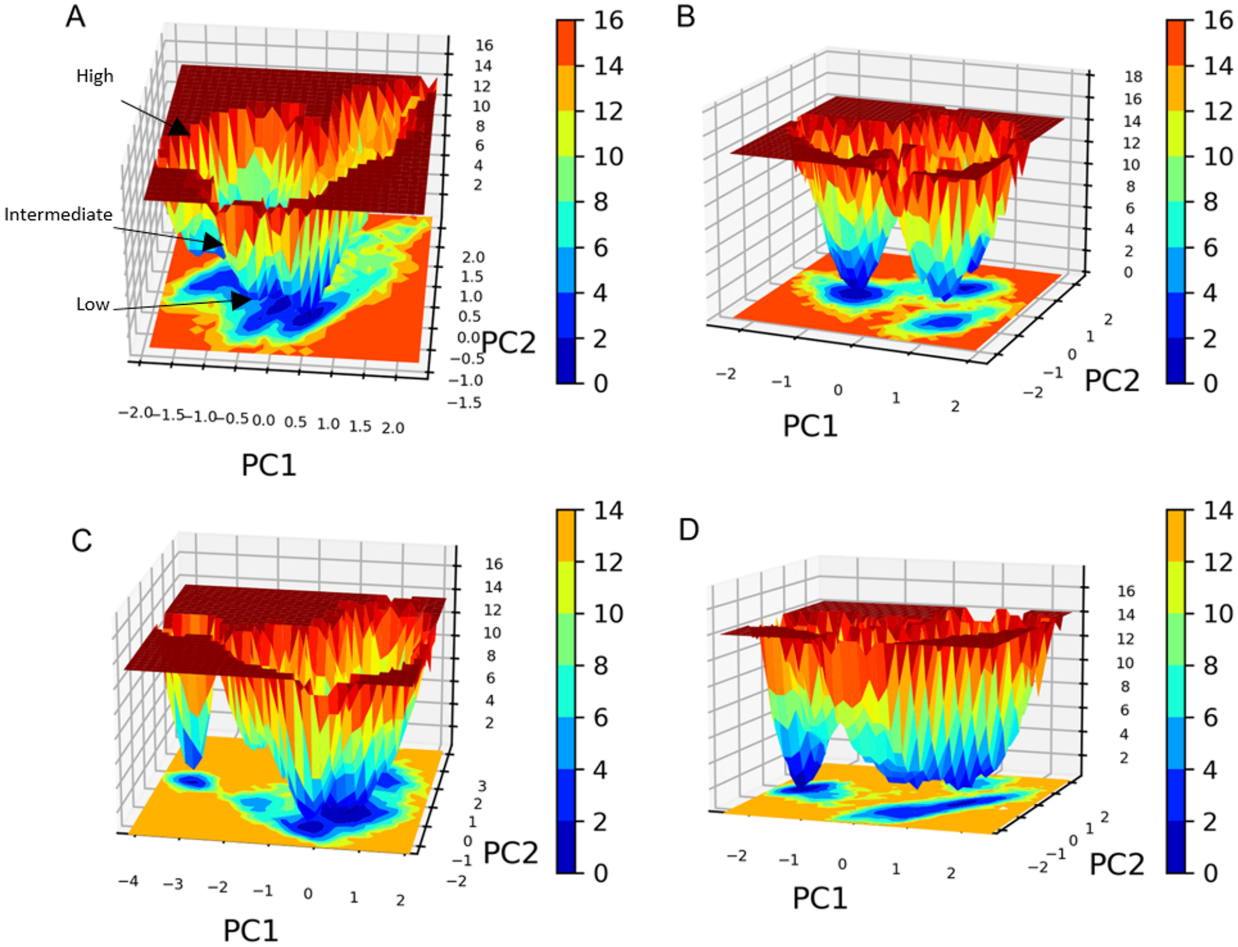
Gibbs free energy 3D landscape. (A) and (B) compound **1**-SARS COV-2 M^pro^ complex during the first and second MD simulations, respectively. (C) and (D) standard (**III**)-SARS COV-2 M^pro^ complex during the first and second MD simulations, respectively.

The free energy landscape (FEL) analysis, which is based on PCA, provides a more accurate depiction of the protein conformational space in terms of energy and time. FEL computation is a computationally practical task; FEL distinguishes the kinetic and thermodynamic attributes of protein holo and apo forms (Al-Khafaji & Taskin Tok, 2020). The FEL results are based solely on the likelihood of occurrence of a specific set of data points, which are then converted to a free energy value *via* a simple relationship (Singh, Somvanshi & Grover, 2019). Accordingly FEL calculations, PCA can give an estimate of the conformational sampling accomplished in an MD ensemble and can describe the sampled conformational landscape (Lambrughi et al., 2012). Assessment of the spatial positioning of the spatial positions of atoms in the system *via* Gibbs FEL analysis showed that both complexes exhibited the formation of metastable formations by crossing multiple energy barriers indicating their high stability (Fig. 11). The size and shape of the minimal energy area (in blue) in the free energy 3D landscape map indicate that all studied complexes were stable. In FEL, energy minima basins exhibiting a canonical shaped energy minima imply the presence of a stable conformation, while the flat ended energy minima basins indicate a lack of minima-energy conformations (Tripathi, Muttineni & Singh, 2013). Accordingly, canonical shaped energy minima exhibited by compound **1**-SARS-CoV-2 M^pro^ complex indicate the stability of the established complex and the formation of a stable conformer.

For all four MD simulations, the MM-PBSA binding free energy was determined using the g _mmpbsa tool by extracting the trajectories of the last nanosecond of each MD simulation (100 frames). This script enables the GROMACS package to determine the total free energy for each complex component, such as the complex’s energy, the energy of the receptor and ligand, *etc*. (Ren et al., 2020). The nonpolar solvation energy is typically calculated using the solvent accessible surface area (SASA) model (Wagoner & Baker, 2004). The free energy of solvation also contains the polar solvation energy (electrostatic) and the nonpolar solvation energy (non-electrostatic) (Kono, Ohtsuki & Abe, 1996). The binding free energy then was computed by deducting the total free energies of the ligand and receptor from the total free energy of the complex. The MM-PBSA calculations showed that while the standard compound showed higher energy in both simulations, compound **1** energy calculations were more reproducible throughout both simulations. The Van der Waals interaction energies (Figs. S18A and S18B) of SARS COV-2 M^pro^-compound **1** and the standard (**III**) complexes further validate this conclusion. The Van der Waals interaction energies calculations show that compound **1** possessed a stable hydrophobic interaction with the SARS COV-2 M^pro^ throughout the whole MD simulation during both runs with an average of −160 KJ/mol. On the other hand, the standard compound (**III**) Van der Waals interaction energies calculations exhibited significant fluctuations throughout the conducted MD simulations. Collectively, the MM-PBSA and protein-ligand interaction energies calculations indicate that compound **1** has a very stable binding to the SARS-CoV-2 M^pro^ when compared to the standard compound (**III**).

The pharmacokinetic properties of compound **1** and the standard compound (**III**) were predicted using the freely accessible Swiss ADME server (Daina, Michielin & Zoete, 2017; Daina & Zoete, 2016). This was carried out due to the capability of a molecule in acting as a drug not being guaranteed by its ability to inhibit the target proteins or enzymes, as demonstrated by the majority of medicines failing during clinical trials due to possess poor ADME characteristics (Ibrahim et al., 2015). One of the important rules used to predict a molecule’s ADME characteristics is the Lipinski rule of five, a rule employed to assess drug likeness (Tian et al., 2011). The results predicted compound **1** to be more suitable from a pharmacokinetic point of view due to the absence of any violations of Lipinski’s rule of 5 as well as its projected promising absorption profile.

## Conclusions

Utilizing a multi-step *in silico* approach, a library of 20 million compounds was subjected to phase screening, predicting potential COVID-19 inhibitors based on their binding affinities for the dimeric form of SARS-CoV-2 M^pro^. These methods included pharmacophore generation, structure-based screening, molecular docking study, molecular dynamics simulations and *in silico* ADME investigation. Compound **1** exhibited higher affinity toward SARS-CoV-2 M^pro^ predict compound **1** to be a potent inhibitor of the SARS-CoV-2 M^pro^. These docking results were verified by carrying out MD simulations which demonstrated that when compared to the standard (**III**), compound **1** exhibited higher stability in the RMSD calculation, comparable RMSF, number of hydrogen bonds, Rg, SASA, PCA, FEL and binding free energy. Combining these results with the favorable ADME characteristics predicted for compound **1** indicate its potential to be a promising lead for developing orally available drug for COVID-19. Accordingly, the next step would be subjecting compound **1** to *in vitro* and *in vivo* tests to assess its biological activity against the SARS-CoV-2 M^pro^ paving the way for the development of novel COVID-19 therapeutic candidate.

## Supplemental Information

10.7717/peerj.14120/supp-1 Supplemental Information 1 Supplementary Figures and Tables.

10.7717/peerj.14120/supp-2 Supplemental Information 2 Compound 1 (LIG) first MD simulation.

10.7717/peerj.14120/supp-3 Supplemental Information 3 Compound 1 (LIG) second MD simulation.

10.7717/peerj.14120/supp-4 Supplemental Information 4 Compound III (standard) first MD simulation.

10.7717/peerj.14120/supp-5 Supplemental Information 5 Compound III (standard) second MD simulation.

10.7717/peerj.14120/supp-6 Supplemental Information 6 Raw data exported from the first molecular dynamics simulation of the ligand with SARs SARS-CoV-2 Main Protease inhibitor.

10.7717/peerj.14120/supp-7 Supplemental Information 7 Raw data exported from the second molecular dynamics simulation of the ligand with SARs SARS-CoV-2 Main Protease inhibitor.

10.7717/peerj.14120/supp-8 Supplemental Information 8 Raw data exported from the first molecular dynamics simulation of the standard compound with SARs SARS-CoV-2 Main Protease inhibitor.

10.7717/peerj.14120/supp-9 Supplemental Information 9 Raw data exported from the second molecular dynamics simulation of the standard compound with SARs SARS-CoV-2 Main Protease inhibitor.

## Additional Information and Declarations

### Data Availability

The following information was supplied regarding data availability: The molecular dynamics raw data files are available in the Supplemental Files.

## References

[ref-1] Abraham MJ, Murtola T, Schulz R, Páll S, Smith JC, Hess B, Lindahl E (2015). GROMACS: high performance molecular simulations through multi-level parallelism from laptops to supercomputers. SoftwareX.

[ref-2] Al-Khafaji K, Taskin Tok T (2020). Molecular dynamics simulation, free energy landscape and binding free energy computations in exploration the anti-invasive activity of amygdalin against metastasis. Computer Methods and Programs in Biomedicine.

[ref-3] Anbarasu K, Jayanthi S (2018). Identification of curcumin derivatives as human LMTK3 inhibitors for breast cancer: a docking, dynamics, and MM/PBSA approach. 3 Biotech.

[ref-4] Bharti R, Shukla SK (2021). Molecules against COVID-19: an in silico approach for drug development. Journal of Electronic Science and Technology.

[ref-5] Bianchi E, Pessi A (2002). Inhibiting viral proteases: challenges and opportunities. Peptide Science.

[ref-6] Cavasotto CN, Lamas MS, Maggini J (2021). Functional and druggability analysis of the SARS-CoV-2 proteome. European Journal of Pharmacology.

[ref-7] Chiaramonte N, Bua S, Ferraroni M, Nocentini A, Bonardi A, Bartolucci G, Durante M, Lucarini L, Chiapponi D, Dei S, Manetti D, Teodori E, Gratteri P, Masini E, Supuran CT, Romanelli MN (2018). 2-Benzylpiperazine: a new scaffold for potent human carbonic anhydrase inhibitors. Synthesis, enzyme inhibition, enantioselectivity, computational and crystallographic studies and in vivo activity for a new class of intraocular pressure lowering agents. European Journal of Medicinal Chemistry.

[ref-8] Choudhury C, Arul Murugan N, Priyakumar UD (2022). Structure-based drug repurposing: traditional and advanced AI/ML-aided methods. Drug Discovery Today.

[ref-9] Daina A, Michielin O, Zoete V (2017). SwissADME: a free web tool to evaluate pharmacokinetics, drug-likeness and medicinal chemistry friendliness of small molecules. Scientific Reports.

[ref-10] Daina A, Zoete V (2016). A boiled-egg to predict gastrointestinal absorption and brain penetration of small molecules. ChemMedChem.

[ref-11] Dhankhar P, Dalal V, Golemi-Kotra D, Kumar P (2020). *In-silico* approach to identify novel potent inhibitors against GraR of *S aureus*. Frontiers in Bioscience.

[ref-12] Drożdżal S, Rosik J, Lechowicz K, Machaj F, Kotfis K, Ghavami S, Łos MJ (2020). FDA approved drugs with pharmacotherapeutic potential for SARS-CoV-2 (COVID-19) therapy. Drug Resistance Updates.

[ref-13] Elhady SS, Abdelhameed RFA, Malatani RT, Alahdal AM, Bogari HA, Almalki AJ, Mohammad KA, Ahmed SA, Khedr AIM, Darwish KM (2021). Molecular docking and dynamics simulation study of hyrtios erectus isolated scalarane sesterterpenes as potential SARS-CoV-2 dual target inhibitors. Biology (Basel).

[ref-14] Elsherbeny MH, Elkamhawy A, Nada H, Abdellattif MH, Lee K, Roh EJ (2021). Development of new meridianin/leucettine-derived hybrid small molecules as nanomolar multi-kinase inhibitors with antitumor activity. Biomedicines.

[ref-15] Gao K, Wang R, Chen J, Tepe JJ, Huang F, Wei G-W (2021). Perspectives on SARS-CoV-2 main protease inhibitors. Journal of Medicinal Chemistry.

[ref-16] Gao Q, Yang L, Zhu Y (2010). Pharmacophore based drug design approach as a practical process in drug discovery. Current Computer-Aided Drug Design.

[ref-17] Greenidge PA, Kramer C, Mozziconacci J-C, Wolf RM (2013). MM/GBSA binding energy prediction on the PDBbind data set: successes, failures, and directions for further improvement. Journal of Chemical Information and Modeling.

[ref-18] Gupta S, Singh AK, Kushwaha PP, Prajapati KS, Shuaib M, Senapati S, Kumar S (2021). Identification of potential natural inhibitors of SARS-CoV2 main protease by molecular docking and simulation studies. Journal of Biomolecular Structure and Dynamics.

[ref-19] Gurung AB, Bhattacharjee A (2018). Met117 oxidation leads to enhanced flexibility of cardiovascular biomarker- lipoprotein- associated phospholipase A2 and reduced substrate binding affinity with platelet-activating factor. International Journal of Biological Macromolecules.

[ref-20] Humphrey W, Dalke A, Schulten K (1996). VMD: visual molecular dynamics. Journal of Molecular Graphics.

[ref-21] Ibrahim HS, Abou-Seri SM, Tanc M, Elaasser MM, Abdel-Aziz HA, Supuran CT (2015). Isatin-pyrazole benzenesulfonamide hybrids potently inhibit tumor-associated carbonic anhydrase isoforms IX and XII. European Journal of Medicinal Chemistry.

[ref-22] Jain SV, Ghate M (2014). Atom-based pharmacophore modeling, CoMFA/CoMSIA-based 3D-QSAR studies and lead optimization of DPP-4 inhibitors for the treatment of type 2 diabetes. Medicinal Chemistry Research.

[ref-23] Jiang L, Sun Q, Li L, Lu F, Liu F (2021). Molecular insights into the inhibitory effect of GV971 components derived from marine acidic oligosaccharides against the conformational transition of Aβ42 monomers. ACS Chemical Neuroscience.

[ref-24] Jusoh N, Zainal H, Abdul Hamid AA, Bunnori NM, Abd Halim KB, Abd Hamid S (2018). In silico study of carvone derivatives as potential neuraminidase inhibitors. Journal of Molecular Modeling.

[ref-25] Klompas M, Baker MA, Rhee C (2020). Airborne Transmission of SARS-CoV-2: theoretical considerations and available evidence. JAMA.

[ref-26] Kono H, Ohtsuki Y, Abe T (1996). Electrostatic free energy of solvation of an arbitrary charge distribution in the block−walker inhomogeneous dielectric. The Journal of Physical Chemistry.

[ref-27] Kumar D, Kumari K, Jayaraj A, Kumar V, Kumar RV, Dass SK, Chandra R, Singh P (2021). Understanding the binding affinity of noscapines with protease of SARS-CoV-2 for COVID-19 using MD simulations at different temperatures. Journal of Biomolecular Structure and Dynamics.

[ref-28] Kumari R, Kumar R, Lynn A (2014). g_mmpbsa—a GROMACS tool for high-throughput MM-PBSA calculations. Journal of Chemical Information and Modeling.

[ref-29] Lambrughi M, Papaleo E, Testa L, Brocca S, De Gioia L, Grandori R (2012). Intramolecular interactions stabilizing compact conformations of the intrinsically disordered kinase-inhibitor domain of Sic1: a molecular dynamics investigation. Frontiers in Physiology.

[ref-30] Laskowski RA, Rullmannn JA, MacArthur MW, Kaptein R, Thornton JM (1996). AQUA and PROCHECK-NMR: programs for checking the quality of protein structures solved by NMR. Journal of Biomolecular NMR.

[ref-31] Lee Y-H, Chatani E, Sasahara K, Naiki H, Goto Y (2009). A comprehensive model for packing and hydration for amyloid fibrils of beta2-microglobulin. Journal of Biological Chemistry.

[ref-32] Leonis G, Czyżnikowska Ż, Megariotis G, Reis H, Papadopoulos MG (2012). Computational studies of darunavir into HIV-1 protease and DMPC bilayer: necessary conditions for effective binding and the role of the flaps. Journal of Chemical Information and Modeling.

[ref-33] Li J, Abel R, Zhu K, Cao Y, Zhao S, Friesner RA (2011). The VSGB 2.0 model: a next generation energy model for high resolution protein structure modeling. Proteins: Structure, Function, and Bioinformatics.

[ref-34] Lokhande KB, Doiphode S, Vyas R, Swamy KV (2021). Molecular docking and simulation studies on SARS-CoV-2 Mpro reveals Mitoxantrone, Leucovorin, Birinapant, and Dynasore as potent drugs against COVID-19. Journal of Biomolecular Structure and Dynamics.

[ref-35] Madhavi Sastry G, Adzhigirey M, Day T, Annabhimoju R, Sherman W (2013). Protein and ligand preparation: parameters, protocols, and influence on virtual screening enrichments. Journal of Computer-Aided Molecular Design.

[ref-36] McDonald IK, Thornton JM (1994). Satisfying hydrogen bonding potential in proteins. Journal of Molecular Biology.

[ref-37] Mittal L, Srivastava M, Kumari A, Tonk RK, Awasthi A, Asthana S (2021). Interplay among structural stability, plasticity, and energetics determined by conformational attuning of flexible loops in PD-1. Journal of Chemical Information and Modeling.

[ref-38] Miyamoto S, Miyake N, Jarskog LF, Fleischhacker WW, Lieberman JA (2012). Pharmacological treatment of schizophrenia: a critical review of the pharmacology and clinical effects of current and future therapeutic agents. Molecular Psychiatry.

[ref-39] Mohammad T, Shamsi A, Anwar S, Umair M, Hussain A, Rehman MT, AlAjmi MF, Islam A, Hassan MI (2020). Identification of high-affinity inhibitors of SARS-CoV-2 main protease: towards the development of effective COVID-19 therapy. Virus Research.

[ref-40] Mortier J, Rakers C, Bermudez M, Murgueitio MS, Riniker S, Wolber G (2015). The impact of molecular dynamics on drug design: applications for the characterization of ligand-macromolecule complexes. Drug Discovery Today.

[ref-41] Mysinger MM, Carchia M, Irwin JJ, Shoichet BK (2012). Directory of useful decoys, enhanced (DUD-E): better ligands and decoys for better benchmarking. Journal of Medicinal Chemistry.

[ref-42] Nada H, Elkamhawy A, Abdellattif MH, Angeli A, Lee CH, Supuran CT, Lee K (2022a). 4-Anilinoquinazoline-based benzenesulfonamides as nanomolar inhibitors of carbonic anhydrase isoforms I, II, IX, and XII: design, synthesis, in-vitro, and in-silico biological studies. Journal of Enzyme Inhibition and Medicinal Chemistry.

[ref-43] Nada H, Lee K, Gotina L, Pae AN, Elkamhawy A (2022b). Identification of novel discoidin domain receptor 1 (DDR1) inhibitors using E-pharmacophore modeling, structure-based virtual screening, molecular dynamics simulation and MM-GBSA approaches. Computers in Biology and Medicine.

[ref-44] Naidoo D, Roy A, Kar P, Mutanda T, Anandraj A (2021). Cyanobacterial metabolites as promising drug leads against the Mpro and PLpro of SARS-CoV-2: an in silico analysis. Journal of Biomolecular Structure and Dynamics.

[ref-45] Pal M, Berhanu G, Desalegn C, Kandi V (2020). Severe acute respiratory syndrome Coronavirus-2 (SARS-CoV-2): an update. Cureus.

[ref-46] Payne S (2017). Family coronaviridae. Viruses.

[ref-47] Rabaan AA, Al-Ahmed SH, Sah R, Tiwari R, Yatoo MI, Patel SK, Pathak M, Malik YS, Dhama K, Singh KP, Bonilla-Aldana DK, Haque S, Martinez-Pulgarin DF, Rodriguez-Morales AJ, Leblebicioglu H (2020). SARS-CoV-2/COVID-19 and advances in developing potential therapeutics and vaccines to counter this emerging pandemic. Annals of Clinical Microbiology and Antimicrobials.

[ref-48] Ren J, Yuan X, Li J, Lin S, Yang B, Chen C, Zhao J, Zheng W, Liao H, Yang Z, Qu Z (2020). Assessing the performance of the g_mmpbsa tools to simulate the inhibition of oseltamivir to influenza virus neuraminidase by molecular mechanics Poisson-Boltzmann surface area methods. Journal of the Chinese Chemical Society.

[ref-49] Salmas RE, Yurtsever M, Durdagi S (2015). Investigation of inhibition mechanism of chemokine receptor CCR5 by micro-second molecular dynamics simulations. Scientific Reports.

[ref-50] Shahbaaz M, Nkaule A, Christoffels A (2019). Designing novel possible kinase inhibitor derivatives as therapeutics against Mycobacterium tuberculosis: an in silico study. Scientific Reports.

[ref-51] Singh A, Somvanshi P, Grover A (2019). Pyrazinamide drug resistance in RpsA mutant (∆438A) of Mycobacterium tuberculosis: dynamics of essential motions and free-energy landscape analysis. Journal of Cellular Biochemistry.

[ref-52] Slynko I, Scharfe M, Rumpf T, Eib J, Metzger E, Schüle R, Jung M, Sippl W (2014). Virtual screening of PRK1 inhibitors: ensemble docking, rescoring using binding free energy calculation and QSAR model development. Journal of Chemical Information and Modeling.

[ref-53] Sneha P, George Priya Doss C, Donev R (2016). Chapter seven – molecular dynamics: new frontier in personalized medicine. Advances in Protein Chemistry and Structural Biology.

[ref-54] Sterling T, Irwin JJ (2015). ZINC 15 – ligand discovery for everyone. Journal of Chemical Information and Modeling.

[ref-55] Stortz CA, Johnson GP, French AD, Csonka GI (2009). Comparison of different force fields for the study of disaccharides. Carbohydrate Research.

[ref-56] Studio DJA (2008). Discovery studio. https://discover.3ds.com/discovery-studio-visualizer-download.

[ref-57] Su H-X, Yao S, Zhao W-F, Li M-J, Liu J, Shang W-J, Xie H, Ke C-Q, Hu H-C, Gao M-N, Yu K-Q, Liu H, Shen J-S, Tang W, Zhang L-K, Xiao G-F, Ni L, Wang D-W, Zuo J-P, Jiang H-L, Bai F, Wu Y, Ye Y, Xu Y-C (2020). Anti-SARS-CoV-2 activities in vitro of Shuanghuanglian preparations and bioactive ingredients. Acta Pharmacologica Sinica.

[ref-58] Surti M, Patel M, Adnan M, Moin A, Ashraf SA, Siddiqui AJ, Snoussi M, Deshpande S, Reddy MN (2020). Ilimaquinone (marine sponge metabolite) as a novel inhibitor of SARS-CoV-2 key target proteins in comparison with suggested COVID-19 drugs: designing, docking and molecular dynamics simulation study. RSC Advances.

[ref-59] Swetha RG, Ramaiah S, Anbarasu A (2016). Molecular dynamics studies on D835N mutation in FLT3—its impact on FLT3 protein structure. Journal of Cellular Biochemistry.

[ref-60] Tian S, Li Y, Wang J, Zhang J, Hou T (2011). ADME evaluation in drug discovery. 9. Prediction of oral bioavailability in humans based on molecular properties and structural fingerprints. Molecular Pharmaceutics.

[ref-61] Tian D, Liu Y, Liang C, Xin L, Xie X, Zhang D, Wan M, Li H, Fu X, Liu H, Cao W (2021). An update review of emerging small-molecule therapeutic options for COVID-19. Biomedicine & Pharmacotherapy.

[ref-62] Tripathi SK, Muttineni R, Singh SK (2013). Extra precision docking, free energy calculation and molecular dynamics simulation studies of CDK2 inhibitors. Journal of Theoretical Biology.

[ref-63] Turner PJCfC, Land-Margin Research OGIoS, and Technology B, OR (2005). XMGRACE, Version 5.1. 19. 2. https://plasma-gate.weizmann.ac.il/Grace/.

[ref-64] Umar AK, Zothantluanga JH, Aswin K, Maulana S, Sulaiman Zubair M, Lalhlenmawia H, Rudrapal M, Chetia D (2022). Antiviral phytocompounds ellagic acid and (+)-sesamin of *Bridelia retusa* identified as potential inhibitors of SARS-CoV-2 3CL pro using extensive molecular docking, molecular dynamics simulation studies, binding free energy calculations, and bioactivity prediction. Structural Chemistry.

[ref-65] Vasina M, Velecký J, Planas-Iglesias J, Marques SM, Skarupova J, Damborsky J, Bednar D, Mazurenko S, Prokop Z (2022). Tools for computational design and high-throughput screening of therapeutic enzymes. Advanced Drug Delivery Reviews.

[ref-66] Wagoner J, Baker NA (2004). Solvation forces on biomolecular structures: a comparison of explicit solvent and Poisson-Boltzmann models. Journal of Computational Chemistry.

[ref-67] Wang R, Lai L, Wang S (2002). Further development and validation of empirical scoring functions for structure-based binding affinity prediction. Journal of Computer-Aided Molecular Design.

[ref-68] Wu C, Liu Y, Yang Y, Zhang P, Zhong W, Wang Y, Wang Q, Xu Y, Li M, Li X, Zheng M, Chen L, Li H (2020). Analysis of therapeutic targets for SARS-CoV-2 and discovery of potential drugs by computational methods. Acta Pharmaceutica Sinica B.

[ref-69] Zoete V, Cuendet MA, Grosdidier A, Michielin O (2011). SwissParam: a fast force field generation tool for small organic molecules. Journal of Computational Chemistry.

