# Supplementary material for: Identification of 1H-purine-2,6-dione derivative as a potential SARS-CoV-2 main protease inhibitor: molecular docking, dynamic simulations, and energy calculations
Source: PeerJ. 2022 Oct 7;10:e14120. doi: 10.7717/peerj.14120 (PMC9549888; doi:10.7717/peerj.14120)
Supplement: Supplemental Information 1 [file peerj-10-14120-s001.docx]

**Supplementary material**

**Identification of 1H-purine-2,6-dione derivative as a potential SARS-CoV-2 Main Protease inhibitor: Molecular docking, dynamic simulations, and energy calculations**

**Guidance – remove this box before submitting!**

Yellow callout boxes provide general notes. Please remove the yellow boxes before submitting. For full guidance see <https://peerj.com/about/author-instructions>

Blue highlighted example text should be replaced or removed with your own information.

| **DO**  –Use clear and grammatically correct English.  –Save as US Letter size format.  –Ensure line numbering is enabled.  –Align text LEFT.  –Ensure title, abstract, and author information matches what is entered online during submission. | **DO NOT**  –Embed ANY figures or tables in the text. Instead, upload a separate file for each on the file uploads page when submitting. Example – If you have 3 figures, then you will upload 3 figure files & be asked to add a figure title for each. See <https://peerj.com/about/author-instructions/#figures> for figure formats. |
| --- | --- |

**Hossam Nada ^1,2^, Ahmed Elkamhawy ^1,3^, Kyeong Lee ^1,*^**

*^1^ Department of Medicinal chemistry, College of Pharmacy, Dongguk University-Seoul, Goyang, Republic of Korea*

*^2^ Pharmaceutical Chemistry Department, Faculty of Pharmacy, Badr University in Cairo 11829, Egypt*

*^3^ Department of Pharmaceutical Organic Chemistry, Faculty of Pharmacy, Mansoura University, Mansoura 35516, Egypt*

**^*^** Corresponding authors:

Kyeong Lee: kaylee@dongguk.edu

**Supplementary Tables and Figures**

**1. Supplementary Figures**

**1.1. Figure S1**: SARS-CoV-2 3CL protease Ramachandran plots

**1.2.** **Figure S2:** (A) Percent of actives found in the ligand-complex based hypothesis. (B) Receiver Operating Characteristic (ROC) plot for the ligand-complex based hypothesis.

**1.3. Figure S3:** 2D interaction patterns of the hit compound **6** in complex with SARS-CoV-2 M^pro^ protease (3CL pro).

**1.4. Figure S4:** 2D interaction patterns of the hit compound **7** in complex with SARS-CoV-2 M^pro^ protease (3CL pro).

**1.5.** **Figure S5:** 2D interaction patterns of the hit compound **8** in complex with SARS-CoV-2 M^pro^ protease (3CL pro).

**1.6. Figure S6:** 2D interaction patterns of the hit compound **9** in complex with SARS-CoV-2 M^pro^ protease (3CL pro).

**1.7. Figure S7:** 2D interaction patterns of the hit compound **10** in complex with SARS-CoV-2 M^pro^ protease (3CL pro).

**1.8. Figure S8:** 2D interaction patterns of the hit compound **11** in complex with SARS-CoV-2 M^pro^ protease (3CL pro).

**1.9. Figure S9:** 2D interaction patterns of the hit compound **12** in complex with SARS-CoV-2 M^pro^ protease (3CL pro).

**1.10. Figure S10:** 2D interaction patterns of the hit compound **13** in complex with SARS-CoV-2 M^pro^ protease (3CL pro).

**1.11. Figure S11:** 2D interaction patterns of the hit compound **14** in complex with SARS-CoV-2 M^pro^ protease (3CL pro).

**1.12. Figure S12:** 2D interaction patterns of the hit compound **15** in complex with SARS-CoV-2 M^pro^ protease (3CL pro).

**1.13. Figure S13. Ligand RMSD calculations.** Blue represents the RMSD of compound **1** (Lig) during the first MD simulation. Red represents the RMSD of compound **1** (Lig) during the second MD run. Magenta represents the RMSD of the standard compound (**III**) during the first MD simulation. Dark green represents the RMSD of the standard compound (**III**) during the second MD simulation.

**1.14. Figure S14.** Hydrogen bonds distribution for all four complexes.

**1.15. Figure S15.** Radius of gyration displayed by compounds **III** and **1** (referred to as Lig) complexes during both MD simulations

**1.16. Figure S16.** SASA calculation of the first MD simulation of compound 1 (Lig) and the standard-SARS-CoV-2 M^pro^ complexes

**1.17. Figure S17.** SASA calculation of the second MD simulation of compound 1 (Lig) and the standard-SARS-CoV-2 M^pro^ complexes

**1.18. Figure S18.** Interaction energies of SARS COV-2 M^pro^-compound **1** and the standard (**III**) complexes during the 1st (A) and 2nd (B) MD simulations.

**2. Supplementary tables**

**2.1. Table S1**. Pharmacophore Test set and their activity against SARS COV-2 main protease.

**2.2. Table S2**. The top 30 hits from the SBVS.


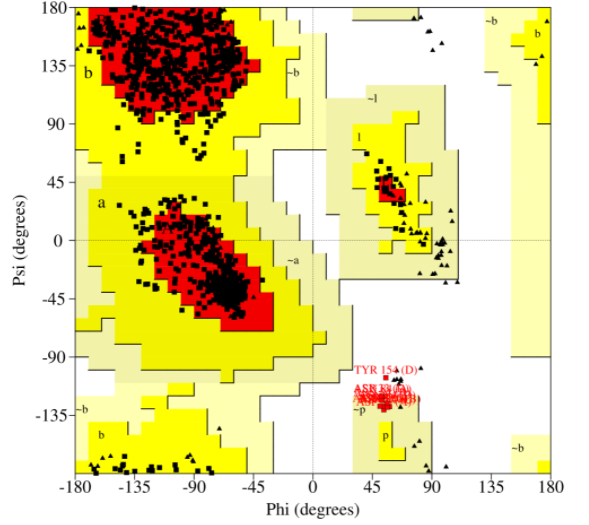


**Figure S1.** SARS-CoV-2 3CL protease Ramachandran plots; the red colored regions indicate the residues present in the most favored region. Additional allowed residues are displayed on a dark yellow background color, additional permitted residues are represented on a light-yellow background color, and disallowed residues are represented on a white background color.


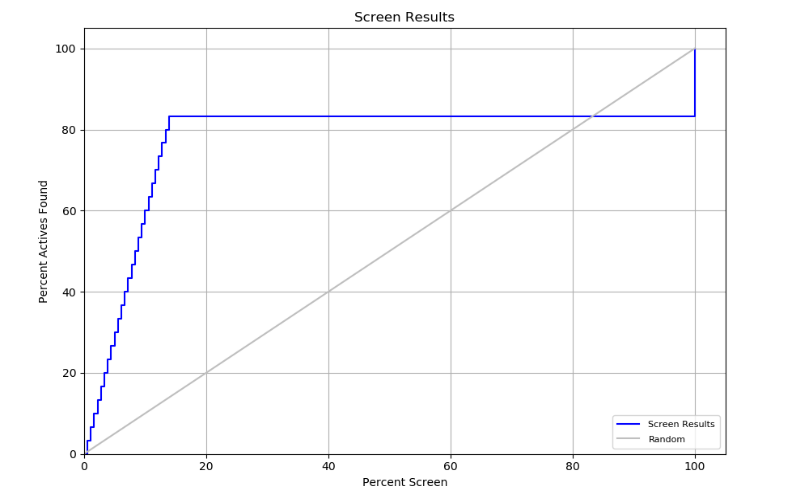

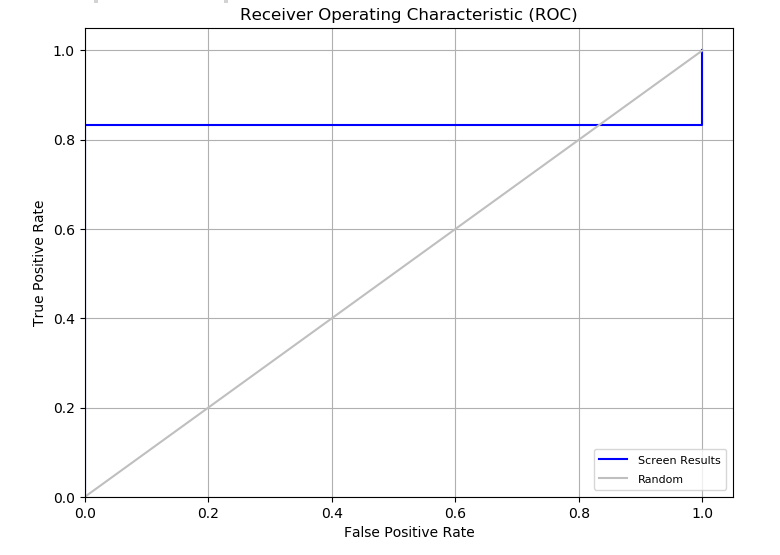


A

B

**Figure S2.** (A) Percent of actives found in the ligand complex-based hypothesis. (B) Receiver Operating Characteristic (ROC) plot for the ligand complex-based hypothesis.


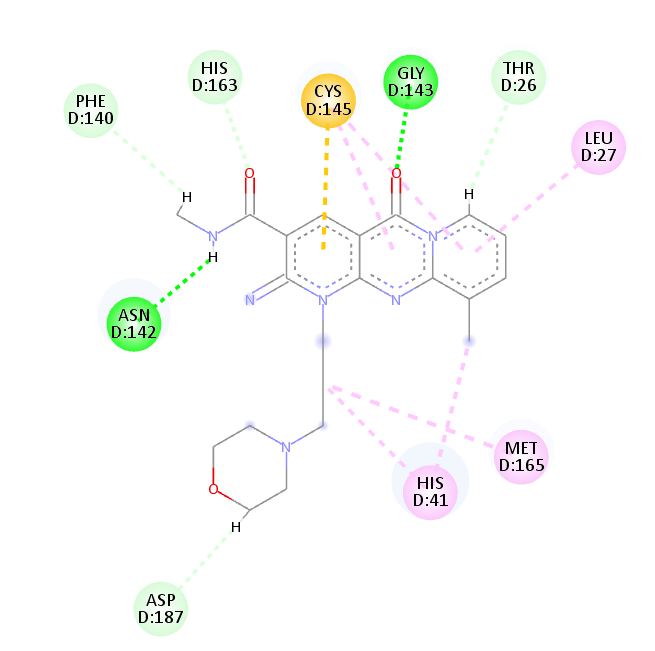


**Figure S3.** 2D interaction patterns of the hit compound **6** in complex with SARS-CoV-2 M^pro^.


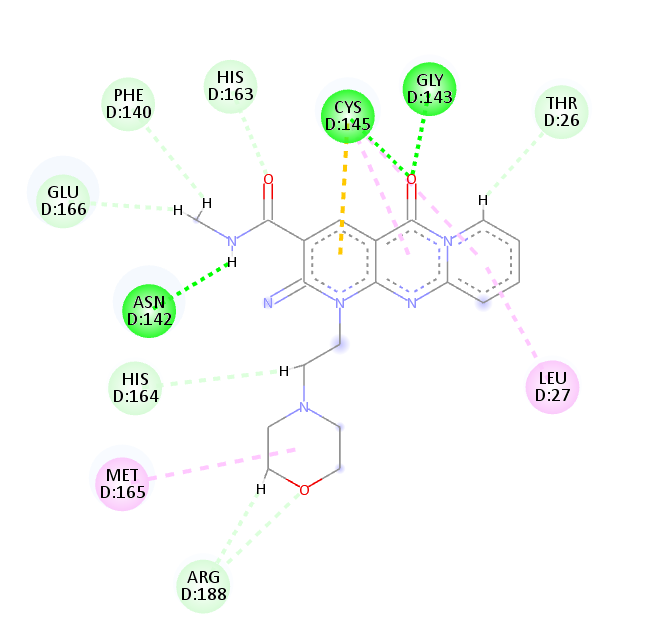


**Figure S4.** 2D interaction patterns of the hit compound **7** in complex with SARS-CoV-2 M^pro^.


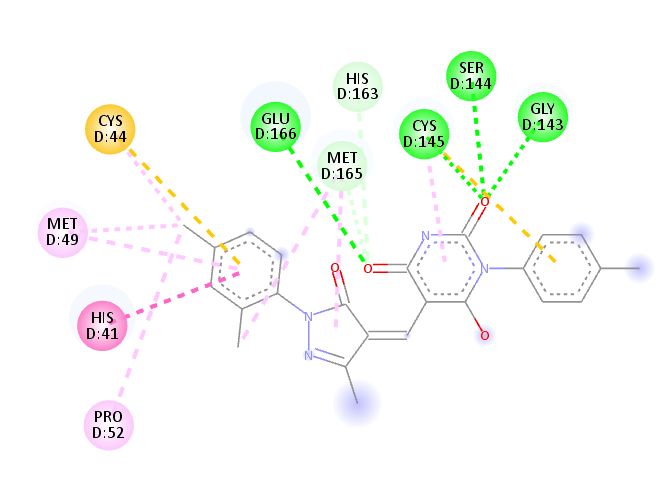


**Figure S5.** 2D interaction patterns of the hit compound **8** in complex with SARS-CoV-2 M^pro^.


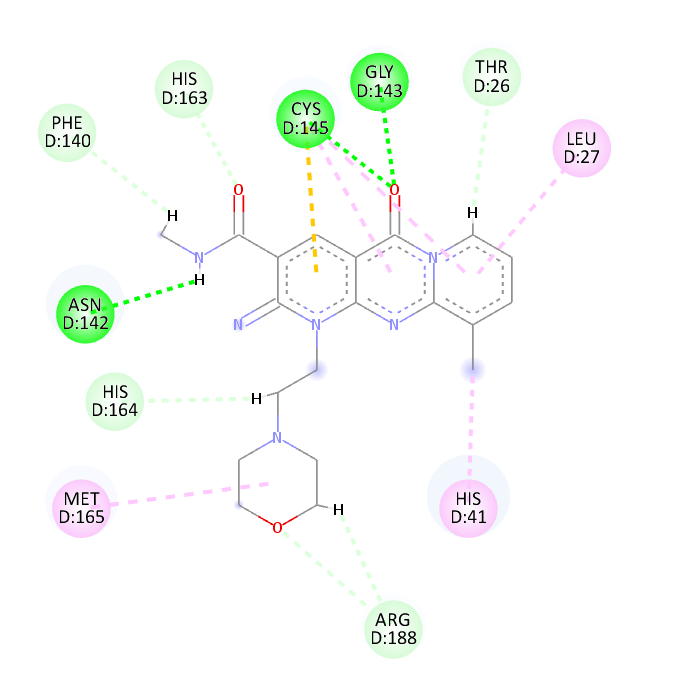


**Figure S6.** 2D interaction patterns of the hit compound **9** in complex with SARS-CoV-2 M^pro^.


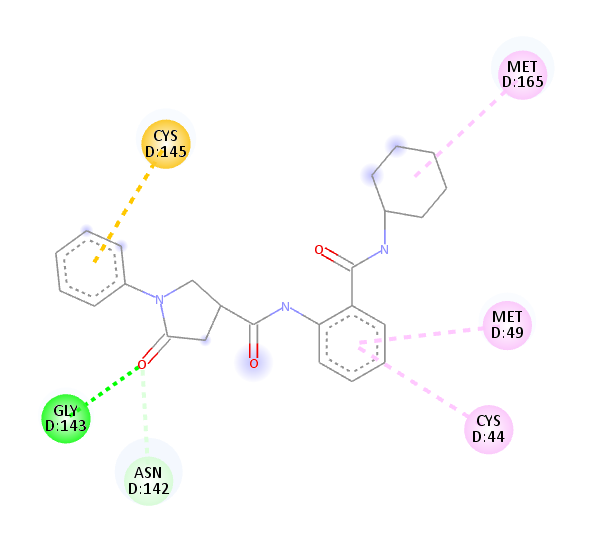


**Figure S7.** 2D interaction patterns of the hit compound **10** in complex with SARS-CoV-2 M^pro^.


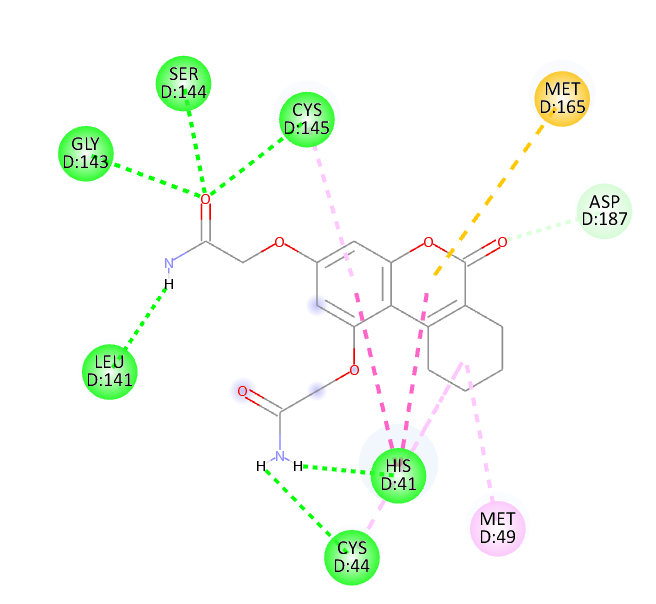


**Figure S8.** 2D interaction patterns of the hit compound **11** in complex with SARS-CoV-2 M^pro^.


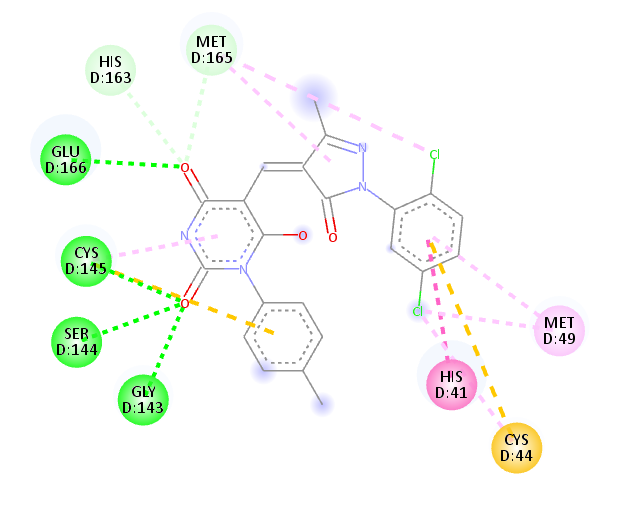


**Figure S9.** 2D interaction patterns of the hit compound **12** in complex with SARS-CoV-2 M^pro^.
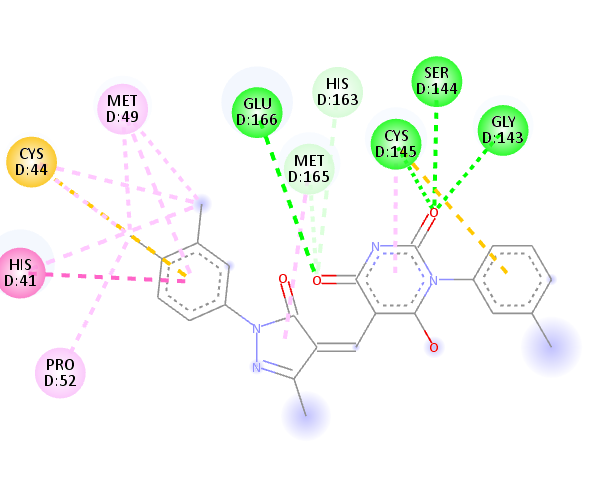


**Figure S10.** 2D interaction patterns of the hit compound **13** in complex with SARS-CoV-2 M^pro^.


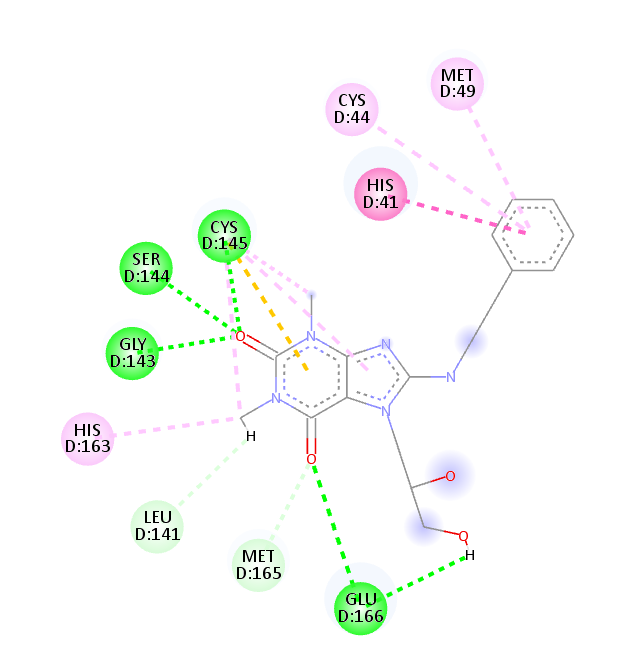


**Figure S11.** 2D interaction patterns of the hit compound **14** in complex with SARS-CoV-2 M^pro^.


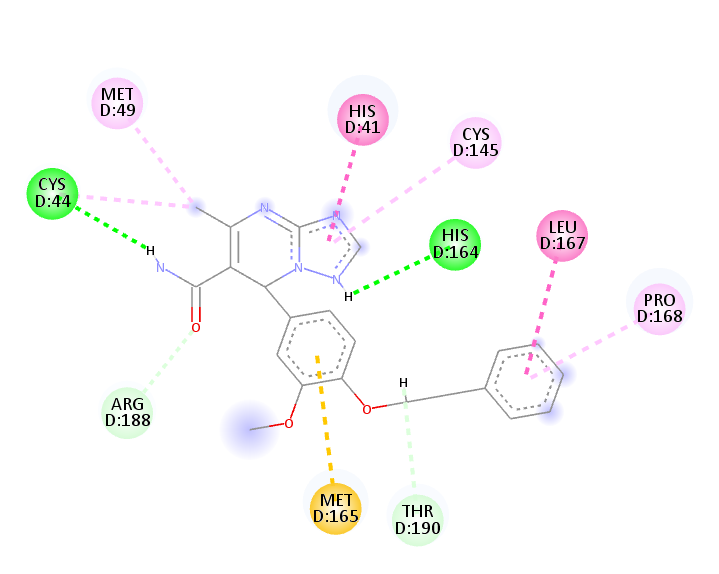


**Figure S12.** 2D interaction patterns of the hit compound **15** in complex with SARS-CoV-2 M^pro^.


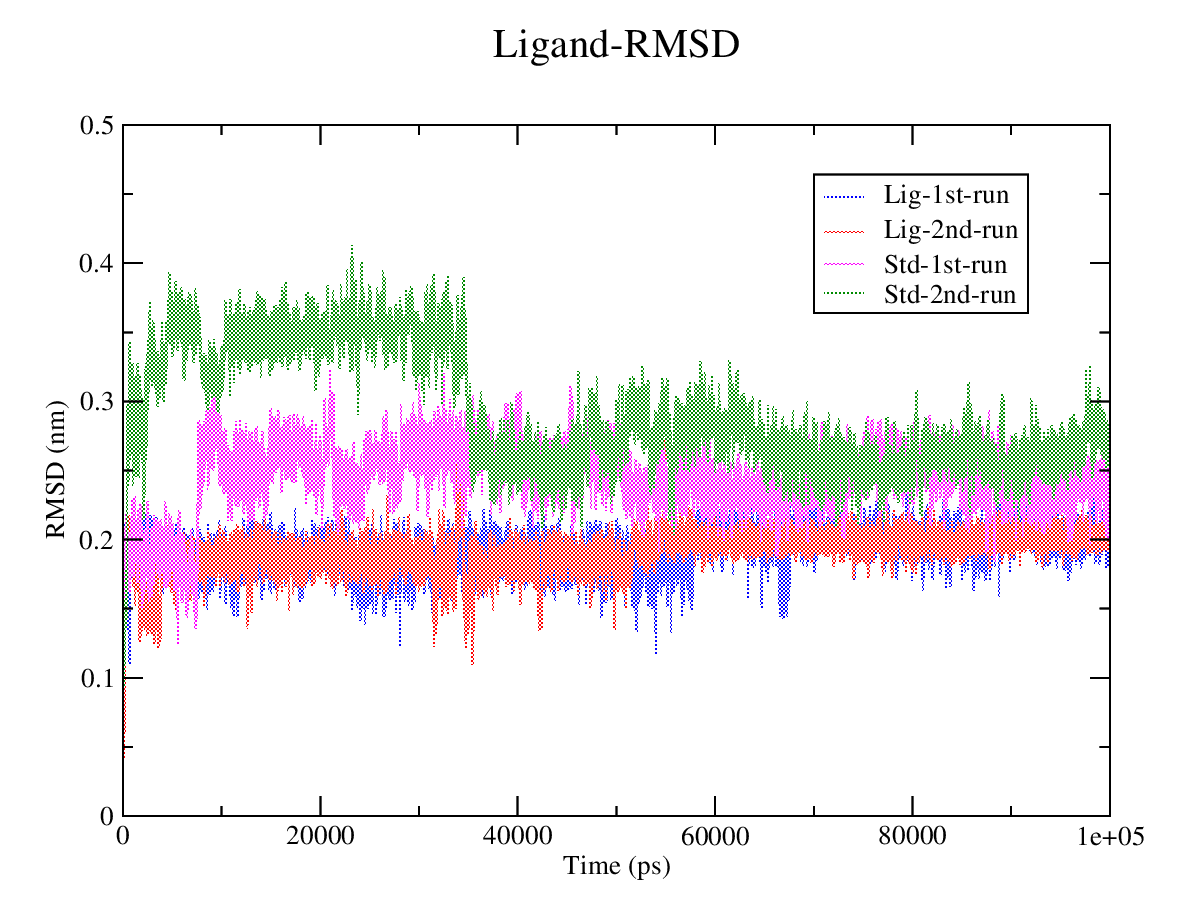


**1.13. Figure S13. Ligand RMSD calculations.** Blue represents the RMSD of compound **1** (Lig) during the first MD simulation. Red represents the RMSD of compound **1** (Lig) during the second MD run. Magenta represents the RMSD of the standard compound (**III**) during the first MD simulation. Dark green represents the RMSD of the standard compound (**III**) during the second MD simulation.


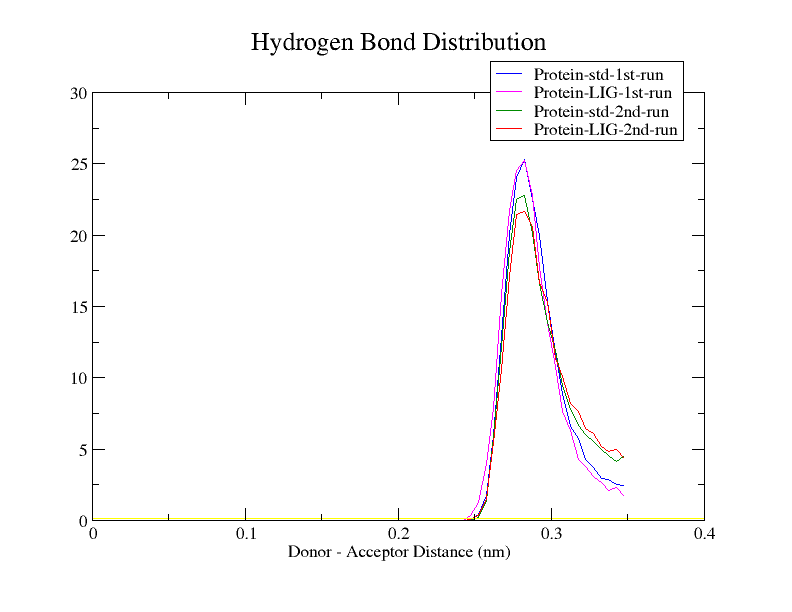


**Figure S14.** Hydrogen bonds distribution for all four complexes.


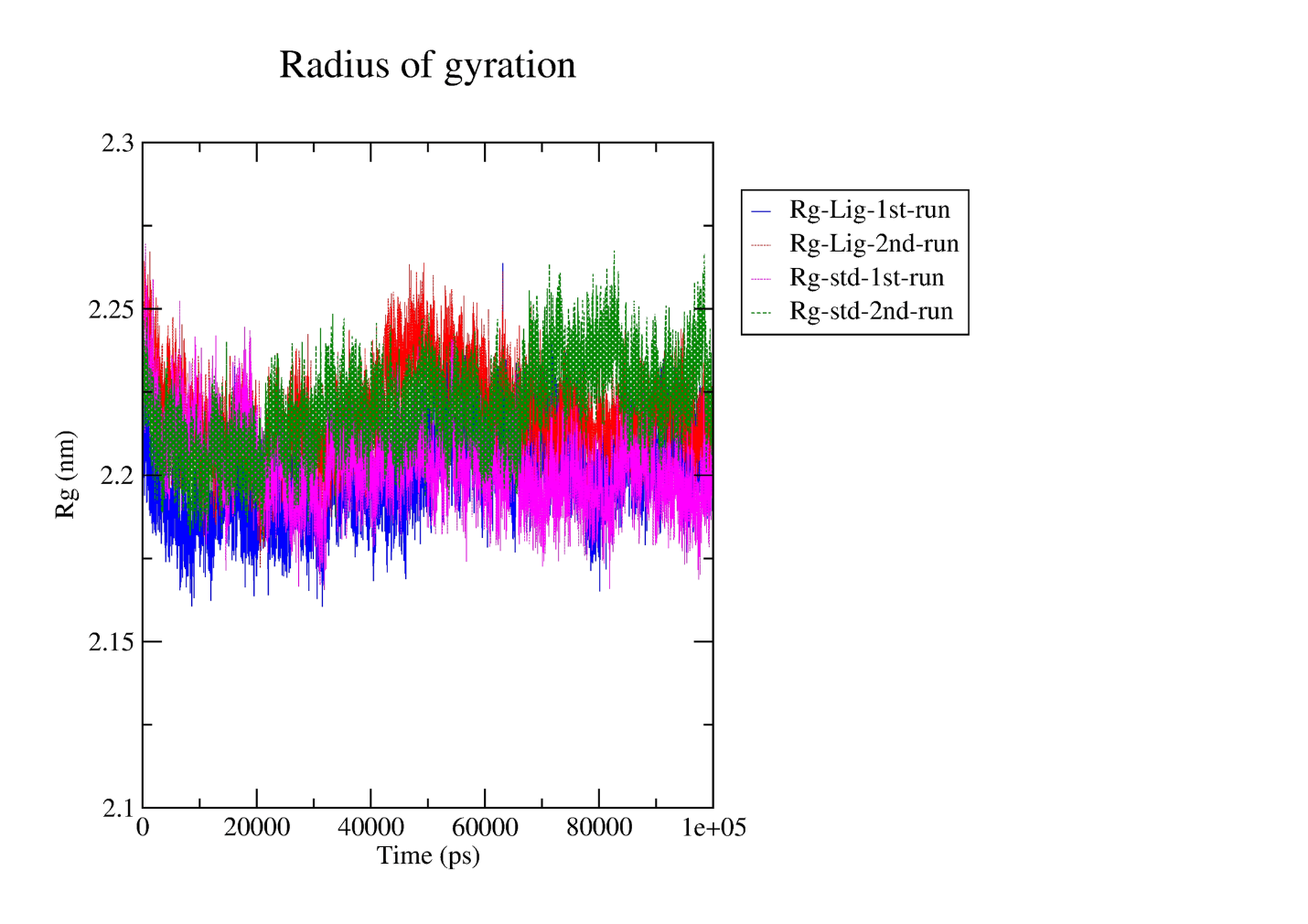


**Figure S15.** Radius of gyration displayed by compounds **III** and **1** (referred to as Lig) complexes during both MD simulations


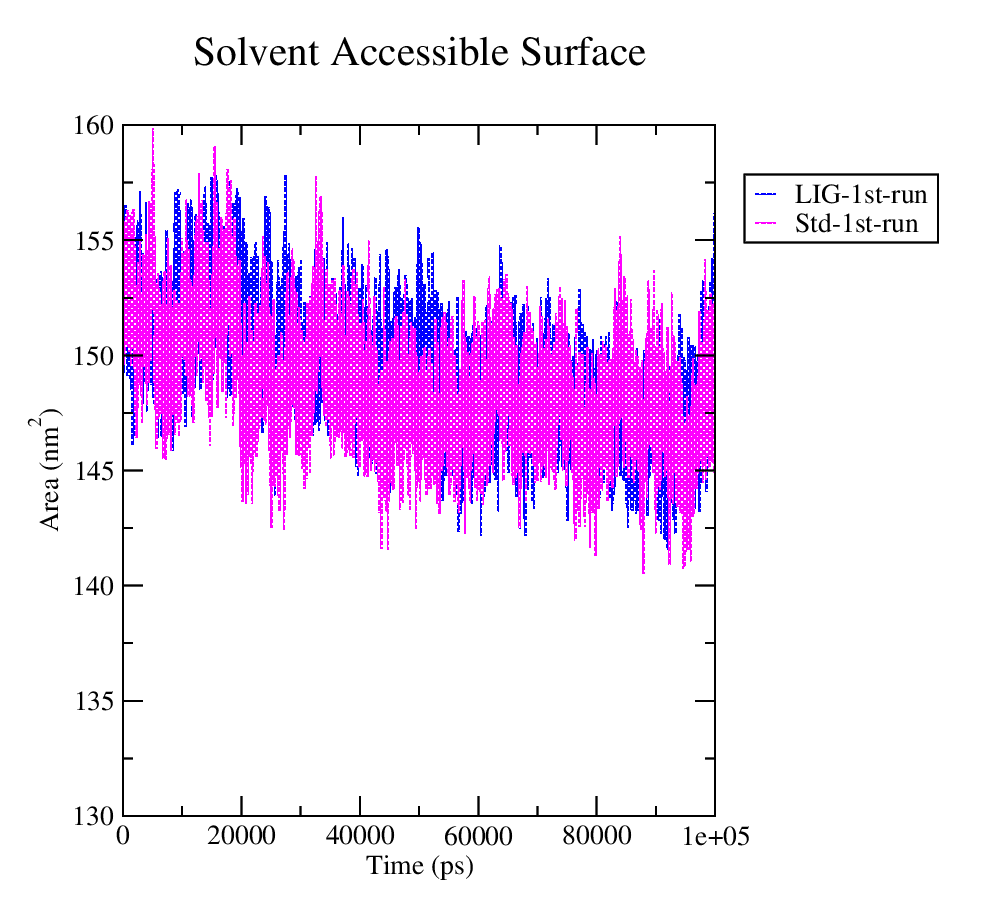


**Figure S16.** SASA calculation of the first MD simulation of compound 1 (Lig) and the standard-SARS-CoV-2 M^pro^ complexes


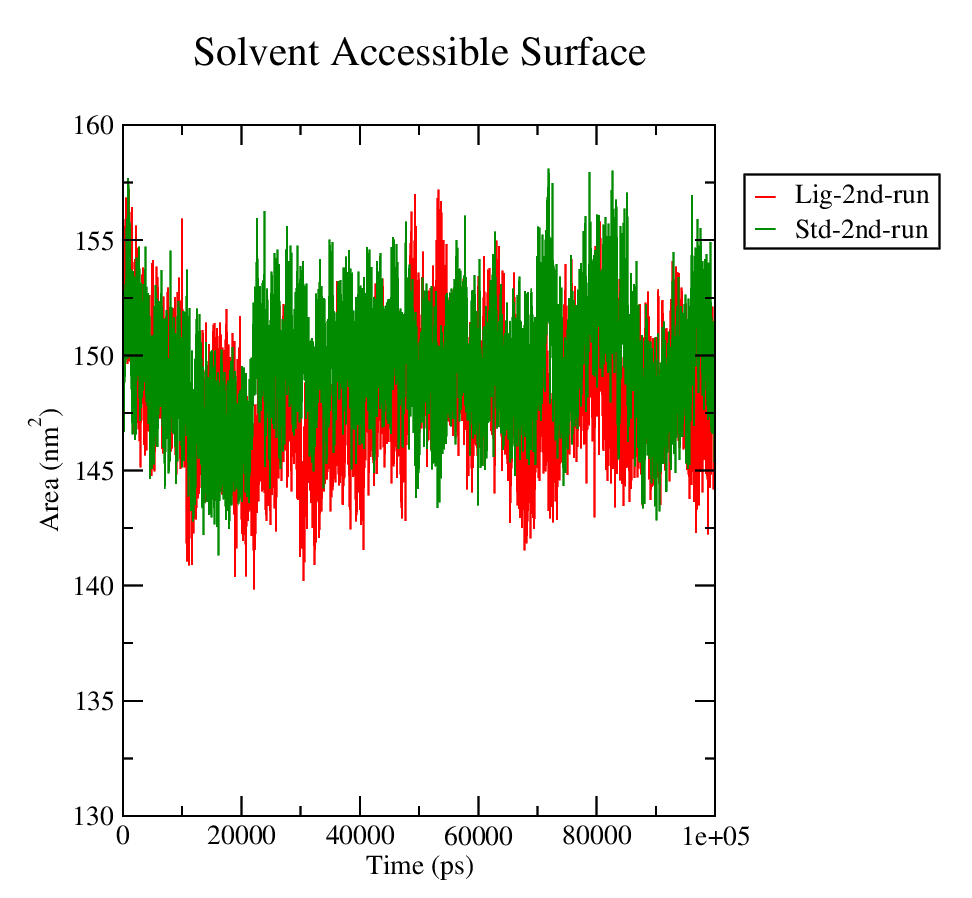


**Figure S17.** SASA calculation of the second MD simulation of compound 1 (Lig) and the standard-SARS-CoV-2 M^pro^ complexes


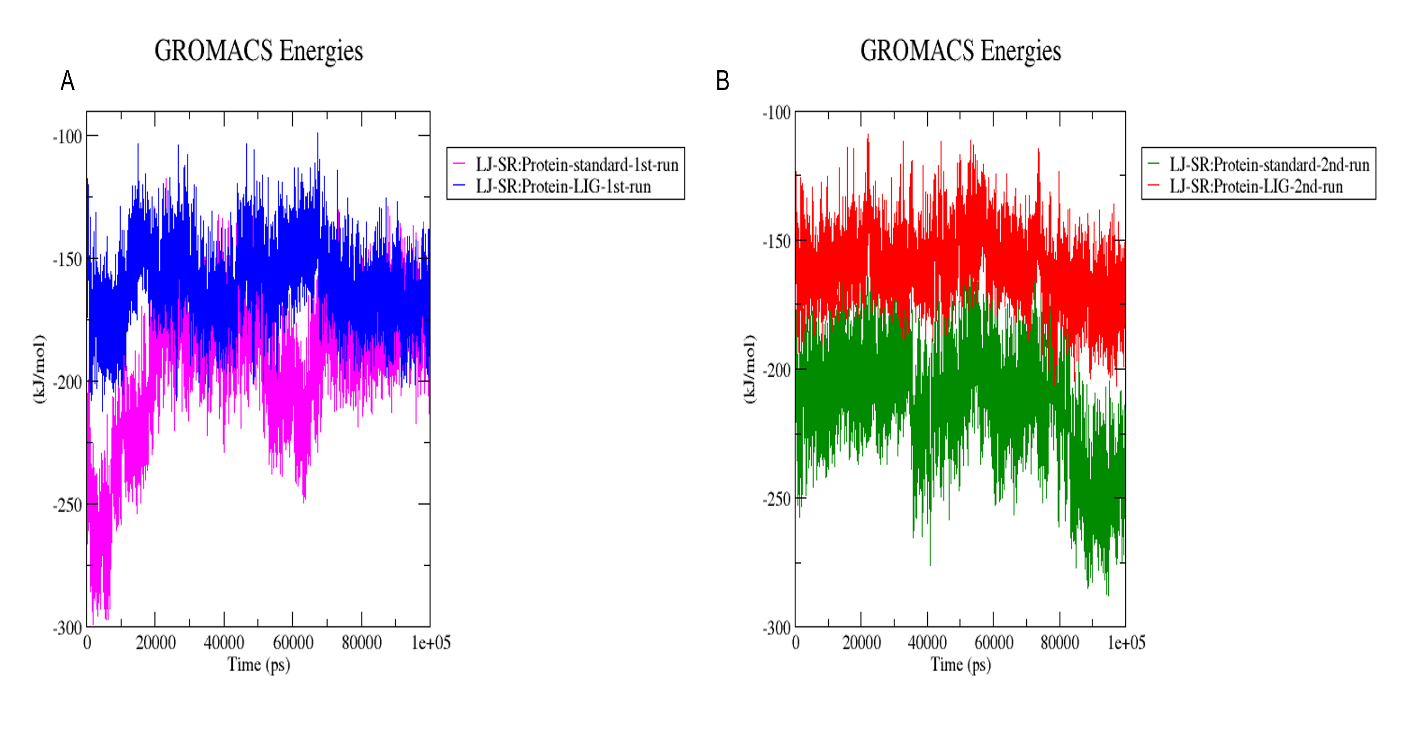


**Figure S18.** Interaction energies of SARS COV-2 M^pro^-compound **1** and the standard (**III**) complexes during the 1st (A) and 2nd (B) MD simulations.

**Table S1.** Pharmacophore Test set and their activity against SARS COV-2 main protease.

| S.N. | Smiles | IC_50_ |
| --- | --- | --- |
| I | O=C1NCC[C@@H]1C[C@H](\C=C\C(=O)OCC)NC(=O)[C@H](Cc2ccc(F)cc2)CC(=O)[C@@H](C(C)C)NC(=O)c3noc(c3)C | 92.55 µM |
| II | CC1=CC(C(NC2=CC=CN([C@@H](CC#C)C(N[C@@H](CC3CCNC3=O)/C=C/C(OCC)=O)=O)C2=O)=O)=NO1 | 68 µM |
| III | c1ccccc1OCC(=O)N[C@@H](C(C)C)C(=O)N[C@@H](CC(C)C)C(=O)N[C@@H](C[C@@H]2CCNC2=O)C(=O)c(n3)sc(c34)cccc4 | 4.1 nM |
| IV | c1cccc(c12)cc([nH]2)C(=O)N[C@@H](CC3CCCCC3)C(=O)N[C@H](C=O)C[C@@H]4CCNC4=O | 0.053 µM |
| V | O=C(c1ccc(C[C@H]2C(N(c3ccc([N+]([O-])=O)cc3)N=C2c4ccccc4)=O)cc1)O | 8.4 µM |
| VI | O=C1NCC[C@@H]1C[C@H](\C=C\C(=O)OCC)NC(=O)[C@H](Cc2ccc(F)cc2)CC(=O)[C@@H](C(C)C)NC(=O)c3noc(c3)C | 0.04 µM |
| VII | c1cc(C(=O)O)ccc1C[C@H]2C(=O)N(c3ccc(C#N)cc3)N=C2c4ccccc4 | 5.5 µM |
| VIII | O=C(O)c1ccc(C[C@H]2C(N(N=C2c3ccccc3)c4ccc(F)cc4)=O)cc1 | 6.8 µM |
| IX | O=C(N[C@H](C(N[C@H](C(N[C@H](C(c1sc2ccccc2n1)=O)C[C@@H]3CCNC3=O)=O)CC(C)C)=O)C(C)C)COc4cccc(C(C)C)c4 | 3 nM |
| X | CC(C)(C)c1ccc(N([C@H](c2cnccc2)C(NC(C)(C)C)=O)C(c3ccco3)=O)cc1 | 1.5 µM |
| XI | c1cc(C(C)(C)C)ccc1N(C(=O)c2occn2)[C@@H](C(=O)NC(C)(C)C)c3cccnc3 | 6 µM |
| XII | CC1=NC(SSC2=CC=CC=C2[N+]([O-])=O)=NC(C)=C1 | 0.92 µM |
| XIII | O=C(N(c1ccc(NC(C2CC2)=O)cc1)[C@H](c3cccn3C)C(NC(C)(CC)C)=O)Cn4nnc5ccccc54 | 9.1 µM |
| XIV | OC1=CC=C(/C=C/C(C2=C(O)C(CC(O)CC)=C(OC)C=C2)=O)C=C1 | 11.4 µM |
| XV | OC1=CC=C(/C=C/C(C2=C(O)C(C/C=C(C)/CCC(O)C(C)C)=C(OC)C=C2)=O)C=C1 | 22.2 µM |
| XVI | BrC1=CC=C(C(C2C(CN[C@H](C([H])=O)CC3=CNC=N3)CC(CCCC4)C4C2)=O)C=C1 | 63 µM |
| XVII | c1cccc(c12)n(nn2)CC(=O)N([C@@H](C(=O)NC(C)(C)CC)c3n(C)ccc3)c4ccc(cc4)NC(=O)CC | 6.9 µM |
| XVIII | c1cccc(c12)n(nn2)CC(=O)N([C@@H](C(=O)NC(C)(C)CC)c3n(C)ccc3)c4ccc(cc4)NC(=O)C5CC5 | 9.1 µM |
| XIX | n1ncoc1SSc2ccc(Cl)cc2 | 0.52 µM |
| XX | Clc1ccc(cc1)SSc2ncc(s2)NC(=O)C | 2.26 µM |
| XXI | Oc1cc(O)cc(c12)C[C@H]([C@H]3C[C@@H](O)[C@H](O)[C@@H](C3)O)[C@@H](C2)OC(=O)c4cc(O)c(O)c(c4)O | 73 µM |
| XXII | Oc1cc(O)cc(c12)C[C@@H]([C@H]3C[C@@H](O)[C@H](O)[C@@H](C3)O)[C@@H](C2)OC(=O)c4cc(O)c(O)c(c4)O | 47 µM |
| XXIII | c1c(O)cc(O)c(c12)c(=O)cc(o2)-c3cc(c(O)cc3)-c(c(O)cc4O)c(c45)oc(cc5=O)-c6ccc(O)cc6 | 8.3 µM |
| XXIV | COc(c1)cc(O)c(c12)c(=O)cc(o2)-c3cc(c(cc3)OC)-c(c(O)cc4O)c(c45)oc(cc5=O)-c6ccc(O)cc6 | 32 µM |
| XXV | CN1C(=O)C(=O)c(c12)cc(cc2)S(=O)(=O)N3CCC(C)CC3 | 1.04 µM |
| XXVI | N1C(=O)C(=O)c(c12)cc(cc2)S(=O)(=O)N3CCC(C)CC3 | 1.18 µM |
| XXVII | c1ccc(C)c(c1C)OCC(=O)N[C@H](Cc2ccccc2)[C@H](O)C[C@@H](Cc3ccccc3)NC(=O)[C@@H](C(C)C)N4CCCNC4=O | 26.63 µM |
| XXVIII | CC(C)c1nc(cs1)CN(C)C(=O)N[C@H](C(C)C)C(=O)N[C@H](Cc2ccccc2)C[C@@H](O)[C@@H](Cc3ccccc3)NC(=O)OCc4cncs4 | 13.7 µM |
| XXIX | CC1COC(C2=C3C(CCCC4(C)CO)C4C=C2)=C1C(C3=O)=O | 24.8 µM |
| XXX | CC1=C(C=CC(C(OCC2C)=C2C3=O)=C4C3=O)C4=CC=C1 | 14.4 µM |

**Table S2.** The top 30 hits from the HTVS

| **Hit** | **Code** | **Number of Sites Matched** | **GlideScore**  **(Kcal/Mol)** |
| --- | --- | --- | --- |
| **1** | ZINC000000920866 | 5 | -8.706 |
| **2** | ZINC000000788680 | 5 | -8.493 |
| **3** | ZINC000000727953 | 5 | -8.49 |
| **4** | ZINC000000788703 | 5 | -8.451 |
| **5** | ZINC000000996474 | 5 | -8.416 |
| **6** | ZINC000000788677 | 5 | -8.349 |
| **7** | ZINC000000727950 | 5 | -8.309 |
| **8** | ZINC000000645906 | 5 | -8.265 |
| **9** | ZINC000000625900 | 5 | -8.236 |
| **10** | ZINC000000043974 | 5 | -8.213 |
| **11** | ZINC000000626346 | 5 | -8.187 |
| **12** | ZINC000000626028 | 5 | -8.179 |
| **13** | ZINC000000838181 | 5 | -8.177 |
| **14** | ZINC000000996257 | 5 | -8.169 |
| **15** | ZINC000000865538 | 5 | -8.132 |
| **16** | ZINC000000936880 | 5 | -8.127 |
| **17** | ZINC000000671420 | 5 | -8.054 |
| **18** | ZINC000000936862 | 5 | -8.028 |
| **19** | ZINC000000688914 | 5 | -8.018 |
| **20** | ZINC000000639429 | 5 | -7.998 |
| **21** | ZINC000000850067 | 5 | -7.984 |
| **22** | ZINC000000846182 | 5 | -7.946 |
| **23** | ZINC000000885282 | 5 | -7.904 |
| **24** | ZINC000000728386 | 5 | -7.895 |
| **25** | ZINC000000999314 | 5 | -7.866 |
| **26** | ZINC000001010581 | 5 | -7.85 |
| **27** | ZINC000000726264 | 5 | -7.813 |
| **28** | ZINC000000677814 | 5 | -7.802 |
| **29** | ZINC000000793655 | 5 | -7.799 |
| **30** | ZINC000000728326 | 5 | -7.762 |
